# γ-Actin plays a key role in endothelial cell motility and neovessel maintenance

**DOI:** 10.1186/s13221-014-0027-2

**Published:** 2015-02-06

**Authors:** Eddy Pasquier, Maria-Pia Tuset, Snega Sinnappan, Michael Carnell, Alexander Macmillan, Maria Kavallaris

**Affiliations:** Tumour Biology and Targeting Program, Children’s Cancer Institute Australia, Lowy Cancer Research Centre, University of New South Wales, P.O. Box 81, 2031 Randwick, NSW Australia; Metronomics Global Health Initiative, Marseille, France; Biomedical Imaging Facility, UNSW, Sydney, Australia; Australian Centre for Nanomedicine, UNSW, Sydney, Australia; Current address: Kolling Institute of Medical Research, Royal North Shore Hospital, 2065 St Leonards, NSW Australia

**Keywords:** Cytoskeleton, Actin, Angiogenesis, Vascular endothelial cells, ROCK signalling

## Abstract

**Background:**

Angiogenesis plays a crucial role in development, wound healing as well as tumour growth and metastasis. Although the general implication of the cytoskeleton in angiogenesis has been partially unravelled, little is known about the specific role of actin isoforms in this process. Herein, we aimed at deciphering the function of γ-actin in angiogenesis.

**Methods:**

Localization of β- and γ-actin in vascular endothelial cells was investigated by co-immunofluorescence staining using monoclonal antibodies, followed by the functional analysis of γ-actin using siRNA. The impact of γ-actin knockdown on the random motility and morphological differentiation of endothelial cells into vascular networks was investigated by timelapse videomicroscopy while the effect on chemotaxis was assessed using modified Boyden chambers. The implication of VE-cadherin, VEGFR-2 and ROCK signalling was then examined by Western blotting and using pharmacological inhibitors.

**Results:**

The two main cytoplasmic isoforms of actin strongly co-localized in vascular endothelial cells, albeit with some degree of spatial preference. While β-actin knockdown was not achievable without major cytotoxicity, γ-actin knockdown did not alter the viability of endothelial cells. Timelapse videomicroscopy experiments revealed that γ-actin knockdown cells were able to initiate morphological differentiation into capillary-like tubes but were unable to maintain these structures, which rapidly regressed. This vascular regression was associated with altered regulation of VE-cadherin expression. Interestingly, knocking down γ-actin expression had no effect on endothelial cell adhesion to various substrates but significantly decreased their motility and migration. This anti-migratory effect was associated with an accumulation of thick actin stress fibres, large focal adhesions and increased phosphorylation of myosin regulatory light chain, suggesting activation of the ROCK signalling pathway. Incubation with ROCK inhibitors, H-1152 and Y-27632, completely rescued the motility phenotype induced by γ-actin knockdown but only partially restored the angiogenic potential of endothelial cells.

**Conclusions:**

Our study thus demonstrates for the first time that β-actin is essential for endothelial cell survival and γ-actin plays a crucial role in angiogenesis, through both ROCK-dependent and -independent mechanisms. This provides new insights into the role of the actin cytoskeleton in angiogenesis and may open new therapeutic avenues for the treatment of angiogenesis-related disorders.

**Electronic supplementary material:**

The online version of this article (doi:10.1186/s13221-014-0027-2) contains supplementary material, which is available to authorized users.

## Background

Angiogenesis is defined as the formation of new blood vessels from pre-existing ones. It is crucial for organ growth during development but also throughout adult life to repair wounded tissues. Furthermore, an imbalance in this process directly contributes to numerous pathologies such as cancer, diabetes, age-related macular degeneration, ischemic disorders and rheumatoid arthritis [1,2]. The multi-step and complex process leading to the formation of a new vascular network relies on the activation of endothelial cells followed by their proliferation, migration and morphological differentiation into capillary tubes. The cytoskeleton which directly regulates and controls an impressive array of cell functions, including cell shape maintenance, cell division, vesicle and organelle transport, cell motility and differentiation, plays a major role in angiogenesis. Studies focusing on the anti-angiogenic properties of microtubule-targeting drugs – reviewed by Pasquier et al. [3] – have provided major insights into the role of microtubules in this process. However, very little is known about the specific role of actin isoforms in angiogenesis.

In vertebrates, there are 6 functional actin genes and the expression of the six actin isoforms is regulated both spatially and temporally in a tissue-specific manner. Four of these isoforms (i.e. α-cardiac muscle actin, α-skeletal muscle actin, α-smooth muscle actin and γ-smooth muscle actin) are mainly expressed in muscle cells, while the cytoplasmic isoforms β- and γ-actin are ubiquitous [4]. Interestingly, the β- and γ-actin isoforms are almost identical proteins, differing only by 4 amino acid residues at the N-terminal end (positions 1, 2, 3 and 9). Distinct localization of β- and γ-actin mRNAs in several cell types, such as neurons, myoblasts and osteoblasts, has suggested for almost 20 years a spatial segregation of the two isoforms [5,6]. However, the spatial and functional segregation of β- and γ-actin was confirmed only recently in fibroblasts and epithelial cells by Chaponnier and colleagues, using newly developed monoclonal antibodies [7]. In particular, β-actin appears to play a role in cell attachment and contraction by preferentially localizing to stress fibres whereas γ-actin is mainly organised as a meshwork in cortical and lamellipodial structures and thus plays a crucial role in cell motility [7]. In accordance with this finding, we recently demonstrated that γ-actin specifically regulates cell motility by modulating the Rho-associated kinase (ROCK) signalling pathway and therefore influencing the phosphorylation of focal adhesion protein paxillin and myosin regulatory light chain 2 in neuroblastoma cells [8]. Elsewhere, key functional differences between β- and γ-actin were also recently revealed by mouse knock-out experiments. Indeed, β-actin knock-out mice are not viable, in part due to severe growth and migration defects of β-actin null embryonic cells, which are not observed in γ-actin null embryonic cells [9]. In contrast, γ-actin knock-out mice are viable, despite suffering increased mortality at birth and progressive hearing loss, which suggests that γ-actin is required for cytoskeleton maintenance but not for development [10]. Spatial segregation and functional differences led us to hypothesize that β- and γ-actin may play distinct roles in endothelial cells and differentially contribute to angiogenesis. We therefore investigated the localization of β- and γ-actin in vascular endothelial cells and undertook the functional analysis of γ-actin by RNAi to decipher its specific function in endothelial cell adhesion, motility and morphological differentiation into vascular networks, thus revealing a key role in angiogenesis.

## Material and methods

### Cell culture

HMEC-1 endothelial cells were originally isolated from dermal microvessels and immortalized by transfection with SV40 large T antigen [11]. They were obtained from the Cell Culture Laboratory in the Hôpital de la Conception (Assistance Publique Hôpitaux de Marseille, Marseille, France) and grown in MCDB-131 medium (Invitrogen, Mount Waverley, Australia) containing 10% heat-inactivated Fetal Calf Serum (FCS), 2 mM L-glutamine, 1% penicillin and streptomycin, 1 μg/mL hydrocortisone and 10 ng/mL epithelial growth factor (BioScientific, Gymea, Australia). BMH29L cells are bone marrow derived endothelial cells that were immortalized by ectopic expression of human telomerase reverse transcriptase [12]. They were kindly provided by Dr Karen MacKenzie (Children’s Cancer Institute Australia) and grown in Medium 199 (Invitrogen) containing 10% heat-inactivated FCS, 5% male human serum AB only (Sigma-Aldrich, Castle Hill, Australia), 1% penicillin and streptomycin, 1% heparin, 5 ng/mL recombinant human FGF_β_ (fibroblast growth factor β; Sigma-Aldrich) and 20 μg/mL Endothelial Cell Growth Factor (ECGF; Roche, Dee Why, Australia). Both cell lines were routinely maintained in culture on 0.1% gelatin-coated flasks at 37°C and 5% CO_2_. Cell lines were regularly screened and are free from mycoplasma contamination.

### Gene silencing

γ-actin gene expression was silenced in endothelial cells using the siRNA sequence previously described (5′-AAGAGATCGCCGCGCTGGTCA-3′; Qiagen, Doncaster, Australia) [13]. An alternative siRNA sequence (5′-CAGCAACACGTCATTGTGTAA-3′; Qiagen) was also used in confirmation experiments [8]. β-actin gene expression was targeted using the siRNA sequence previously described (5′-AATGAAGATCAAGATCATTGC-3′; Qiagen) [14]. The optimum amount of siRNA was determined to be 200 and 500 pmol for HMEC-1 and BMH29L cells, respectively and was used in all subsequent experiments. A non-silencing control siRNA, which has no sequence homology to any known human gene sequence, was used as a negative control in all experiments (Qiagen). Cells were transfected using the Nucleofector® II device (Lonza, Mount Waverley, Australia) as previously described [15]. Briefly, HMEC-1 and BMH29L cells were resuspended in nucleofector® solution R and V, respectively, and transfected with siRNA using specifically optmized nucleofector® programs (T-016 and S-003 for HMEC-1 and BMH29L, respectively). All subsequent experiments were performed 72 h after siRNA transfection, when the level of γ-actin protein expression was the lowest.

### Quantitative RT-PCR

The expression of γ-actin mRNA was examined using quantitative RT-PCR. Total RNA was extracted and DNase treated using the Qiagen RNeasy Plus kit according to the manufacturer’s instructions (Qiagen) and cDNA synthesis was performed using High capacity cDNA reverse transcription kit with RNAse inhibitor (Applied Biosystems, Mulgrave, Australia). Real-time PCR was performed on 7900HT Fast Real-time PCR system using the TaqMan® gene expression Master Mix (Applied Biosystems). γ-actin mRNA primer and probe sequences used were as follows: forward, 5′-CAGCTCTCGCACTCTGTTCTTC-3′; reverse, 5′-ACATGCCGGAGCCATTGT-3′; probe, 5′-CGCGCTGGTCATT-3′. All data were normalized to the housekeeping gene *Ppia* (peptidilprolyl isomerase A, TaqMan® Endogenous Control, Applied Biosystems). Gene expression levels were determined using the ΔΔ*C*_t_ method, normalized to the housekeeper gene and expressed relative to a calibrator [16].

### Western blotting analysis

For western blotting analysis, cells were lysed in RIPA buffer containing a cocktail of protease and phosphatase inhibitors (Sigma-Aldrich). Equal amounts of protein (10–15 μg) were resolved on 12% sodium dodecyl sulfate polyacrylamide gel electrophoresis or 4-15% pre-cast Criterion acrylamide gels (Bio-Rad Laboratories, Gladesville, Australia) before electrotransfer onto nitrocellulose membrane. Immunoblotting was performed using antibodies directed against β-actin (clone AC-74, Sigma-Aldrich), γ-actin – courtesy of Pr Peter Gunning [17], GAPDH (Abcam, Cambridge, UK), phospho-myosin light chain 2 (Cell Signaling Technology, Beverly, MA, USA), VE-cadherin (Cell signaling technology) and VEGFR-2 (Cell signaling technology). The membranes were then incubated with horseradish peroxidase-conjugated IgG secondary antibodies and protein detected with ECL Plus (GE Healthcare Life Sciences, Uppsala, Sweden). The blots were scanned and densitometric analysis performed as previously described [13].

### Immunofluorescence staining

Vascular endothelial cells were seeded on gelatin-coated 8-well Permanox Lab-Tek chamber slides (Applied Biosystems) after siRNA transfection. β-actin and γ-actin were stained as previously described [7] with slight modifications. Specifically, cells were fixed with 3.7% formaldehyde for 20 min at RT and permeabilized with 100% methanol for 20 min at −20°C. Cells were incubated with the following primary mAbs: anti-β-actin (mAb 4C2, IgG1 – courtesy of Pr Christine Chaponnier [7]) and anti-γ-actin (mAb 2A3, IgG2b – courtesy of Pr Christine Chaponnier [7]). The following secondary Abs were used: FITC-conjugated goat anti-mouse IgG1 (Southern Biotechnology, Birmingham, AL) and TRITC-conjugated goat anti-mouse IgG2b (Southern Biotechnology). For tubulin staining, cells were fixed and permeabilized in 100% methanol at 20°C for 15 min and blocked with 10% FCS for 30 min. Microtubules were then stained with anti-βI-tubulin primary antibody (Abcam), followed by Alexa Fluor 488 anti-mouse secondary antibody (Invitrogen). For paxillin and phalloidin dual staining, cells were fixed with 3.7% formaldehyde/PBS for 10 min and permeabilized with 0.1% Triton X-100/PBS for 5 min. Focal adhesions were then stained with anti-paxillin primary antibody (BD Biosciences), followed by Alexa Fluor 488 anti-mouse secondary antibody (Invitrogen) and Alexa 568-conjugated phalloidin (Invitrogen). All slides were mounted on coverslips with ProLong Gold anti-fade reagent containing DAPI (Invitrogen) and imaged using the 63X oil-immersion objective of an Axiovert 200 M fluorescent microscope coupled to an AxioCamMR3 camera driven by the AxioVision 4.8 software (Carl Zeiss, North Ryde, Australia). The thickness of actin stress fibres was determined performing a line scan perpendicular to the fibres using Image J, while the size of paxillin-containing adhesion sites was measured using the AxioVision 4.8 software.

### Colocalization analysis

Colocalization between β-actin and γ-actin channels was assessed by measuring the Pearson’s Correlation Coefficient and visually inspecting two-dimensional histograms (fluorograms). The Pearson’s coefficient measures the linear relationship between the pixel intensities of two channels. In the case of positive correlation this value can drop either due to decreasing colocalization, or due to differences in stoichiometry in structures. The fluorogram can distinguish these two scenarios, low colocalization shows by dispersion of points whilst varying stochiometries show multiple tight linear clusters. Measurements were made using the Coloc 2 plugin in ImageJ (imagej.nih.gov/ij). Image background was carefully subtracted from each channel and regions of interest were drawn around each cell to exclude extracellular pixels from the measurement. Measurements were performed on single cells to ensure variations in expression and staining did not contribute to multiple stoichiometries.

### Adhesion assay

For the adhesion assay, cells were pre-labeled *in situ* with 10 μM Cell Tracker Green CMFDA (Invitrogen) in serum-free medium for 30 min and 50,000 cells were then seeded onto 24-well plates, pre-coated for 2 hours at 37°C with various extra-cellular matrix (ECM) proteins: fibronectin (2 μg/mL), laminin (10 μg/mL) or type I collagen (10 μg/mL). After 1 hour incubation, cells were washed twice with PBS and the number of adhered cells was assessed with a Victor 3 plate reader (Perkin-Elmer, Glen Waverley, Australia) at 492/517 (Abs/Em). All readings were then normalized to the negative control (no ECM).

### Chemotaxis assay

The chemotaxis assay was performed as previously described [18]. Briefly, the underside of 8 μm transparent polyethylene terephthalate membrane inserts (BD Falcon) was pre-coated with 0.1% gelatin for 1 h. The cells were pre-labeled *in situ* with 10 μM Cell Tracker Green CMFDA (Invitrogen) in serum-free medium for 30 min and 100,000 cells were then seeded onto the insert in assay medium (0.5% BSA in serum-free medium). Assay medium supplemented with 5% FCS, 0.1 ng/mL VEGF-A, 5 ng/mL FGF_β_ or 20 μg/mL ECGF was then added to the bottom of the insert and used as chemoattractant. A negative control was included in each experiment by adding serum-free medium to the bottom of the insert. The plates were incubated for 6 h at 37°C and 5% CO_2_. Excess cells on the upper side of the insert were then gently swabbed off with a cotton tip and migrated cells at the underside of the insert were measured with the same plate reader used for the adhesion assay. All readings were then normalized to the negative control (serum-free medium).

### Random motility assay

Random cell motility was assessed by time-lapse microscopy as previously described [18]. Briefly, cells were seeded on a 24-well gelatin-coated plate and allowed to adhere for 1 h. Photographs were then taken every 5 min for 6 h in at least 2 view fields per well using the 5X objective of the same microscope device used for immunofluorescence experiments. During this assay, cells were constantly maintained at 37°C and 5% CO_2_. Analysis was performed using the tracking module of the AxioVision 4.8 software. At least 25 cells per view field were tracked for 6 h; cells undergoing division or apoptosis were excluded from analyses. The persistent random-walk model was used to characterize cell motility [19]. For each individual cell, the mean square displacement < D^2^ > was calculated from the following formula:

$$ <{D}^2>={\displaystyle \sum_{i=1}^M{d}_i^2} $$

where d_i_ is the displacement of a cell from its initial position at time level t_i_. The persistence time (P) (i.e. average time interval between significant movements and direction changes) and random motility coefficient (μ) (i.e. the rate at which a cell population is able to migrate into and colonize a new area) were deduced from the < D^2^ > value and the cell velocity (S), using the following formulas:

$$ \begin{array}{c}\hfill <D>=2{\mathrm{S}}^2{\mathrm{P}}^2\left[\left(1/\mathrm{P}\right)-1+ \exp \left(-1/\mathrm{P}\right)\right]\hfill \\ {}\hfill \upmu =\left(1/2\right){\mathrm{S}}^2\mathrm{P}\hfill \end{array} $$

### Wound healing assay

An optimized wound healing assay was used as previously described [20], with slight modifications. Endothelial cells were grown to confluence in specific culture inserts (Ibidi, Martinsried, Germany). After 24 h, the culture inserts were removed, leaving a definite cell-free gap of approximately 400 μm, and the cells were washed with PBS before their incubation in culture medium. The colonization of the cell-free gap was analysed by time-lapse videomicroscopy using the 5X objective of the same microscope device used for immunofluorescence experiments. Photographs were taken every 10 minutes for 20 h and plates were kept at 37°C and 5% CO_2_ throughout the duration of the experiment. The migration rate was calculated digitally by quantification of the cell-free area at the different time points using the AxioVision 4.7 software.

### *In vitro* Matrigel™ assay

Matrigel™ (BD Biosciences, North Ryde, Australia) assay was used to determine the effect of γ-actin knockdown on endothelial cell morphogenesis into capillary tubes, as previously described [18]. Briefly, 24-well plates were coated at 4°C with 270 μL of a Matrigel™ solution (1:1 dilution in culture medium), which was then allowed to solidify for 1 h at 37°C before cell seeding. Cells were allowed to undergo morphogenesis and form capillary-like structures and photographs were taken after 8 h using the 5X objective of the same microscope device used for immunofluorescence experiments. Angiogenesis was then quantitatively evaluated by measuring the total surface area of capillary tubes formed in at least 10 view fields per well using the AxioVision 4.7 software.

A non-enzymatic methodology was also established to analyse the potential changes in protein expression that occur during the morphological differentiation of endothelial cells into vascular networks. Briefly, 3.2 × 10^5^ cells were seeded onto 6-well plates previously coated with Matrigel™ and harvested at different time points of the morphological differentiation process (i.e. 15 min, 1, 2, 4 and 8 h). Cells were first incubated with a Cell Recovery Solution (BD Biosciences) for 1 h at 4°C under agitation to allow complete dissolution of the Matrigel™, then pelleted, washed with cold PBS and finally lysed as described in the western blotting section.

### Rho-associated kinase (ROCK) signalling inhibition

ROCK signalling was interrupted as previously described [8], through the use of two specific ROCK inhibitors, H-1152 (Merck Millipore, Kilsyth, Australia) and Y-27632 (Sigma-Aldrich). Stock solutions of both inhibitors were prepared in water and stored at 4°C. Inhibitors (1–10 μM) were added to siRNA-transfected cells at 48 h post-transfection, and remained in culture medium for a further 24 h and during wound-healing and angiogenesis assays.

### Statistical analysis

All experiments were performed at least in triplicate. Statistical significance was determined using two-sided student’s t test in the GraphPad Prism 4 software (GraphPad Software, Inc).

## Results

### Spatial distribution of β- and γ-actin in vascular endothelial cells

**Figure 1:**
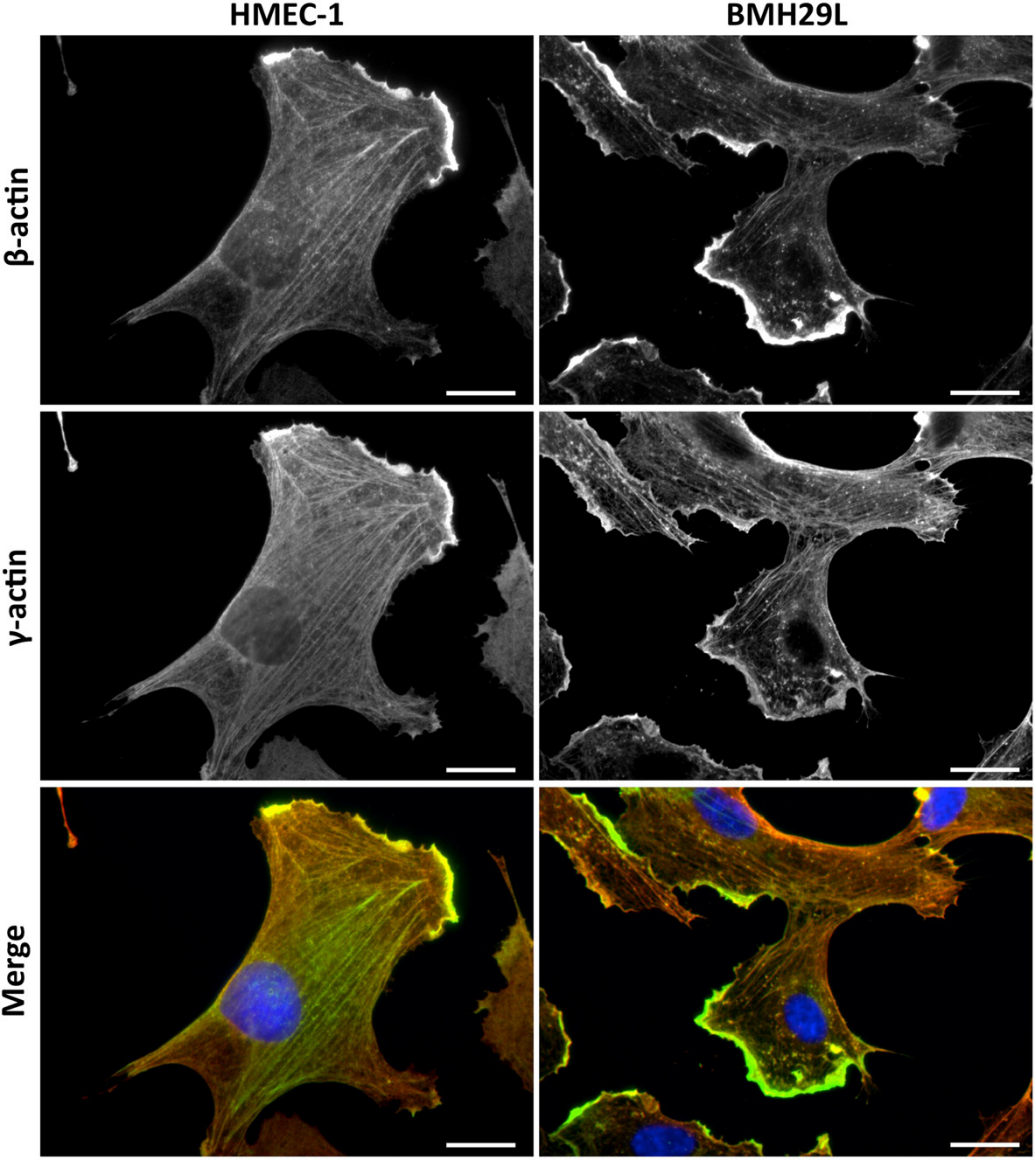
**Localization of** **β- and** **γ-actin in vascular endothelial cells.** Representative photographs of HMEC-1 (*left*) and BMH29L (*right*) endothelial cells stained with β-actin (*top*) and γ-actin (*middle*) antibodies. The merged photographs (*bottom*) show β-actin in green, γ-actin in red and DNA (DAPI) in blue. *Scale bar*, 20 μm.

Using specific monoclonal antibodies directed against β- and γ-actin [7], we investigated the cellular distribution of both actin isoforms in two models of vascular endothelial cells (Figure 1). These co-immunofluorescence experiments demonstrated extensive colocalization of the two actin isoforms with some level of spatial preference. In HMEC-1 and BMH29L endothelial cells, β- and γ-actin signals strongly overlapped but β-actin signal appeared relatively more enriched in radial stress fibres and membrane ruffling in relation to γ-actin, which was more uniformly spread across the entire microfilament meshwork. Quantification using the Pearson’s Correlation Coefficient method [21] confirmed the strong colocalization of β- and γ-actin (Additional file 1: Figure S1A). Furthermore, decreased correlation in some cells was mostly due to variations in stoichiometry in different subcellular structures rather than complete segregation of the two actin isoforms (Additional file 1: Figure S1B).

### Knockdown of cytoplasmic γ-actin expression by RNAi

**Figure 2:**
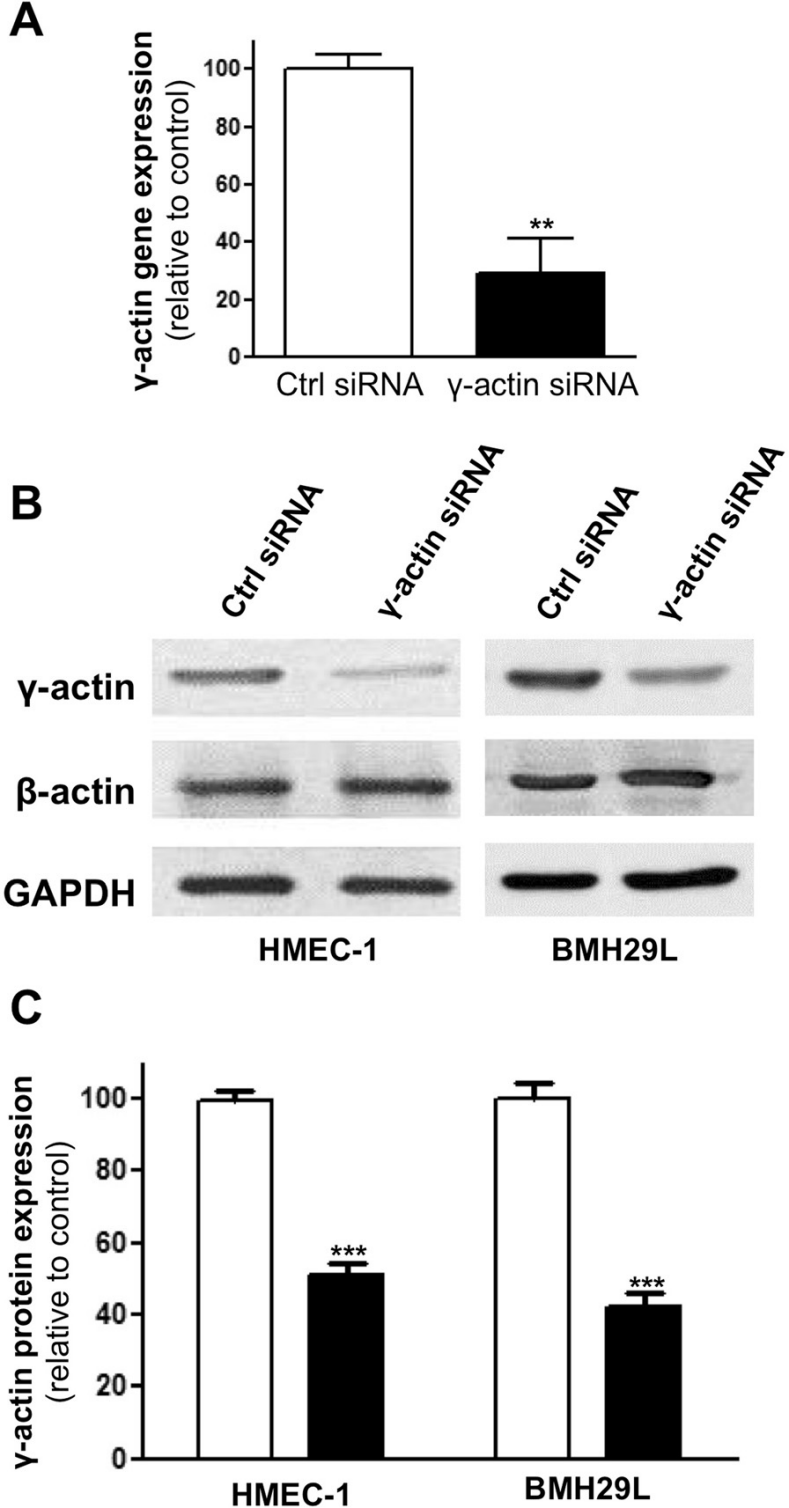
**γ-actin knockdown in vascular endothelial cells. (A)** Histogram showing γ-actin relative gene expression following treatment with control (*white*) and γ-actin siRNA (*black*) for 24 h as assessed by quantitative RT-PCR. β*2-microglobulin* was used as housekeeping gene. *Columns*, means of at least three individual experiments; *bars*, SE. Statistics were calculated by comparing γ-actin relative expression in control and γ-actin siRNA-treated HMEC-1 cells; **, p < 0.01. **(B)** Representative immunoblots of HMEC-1 (*left*) and BMH29L (*right*) cell lysates following treatment with control and γ-actin siRNA for 72 h. Membranes were probed with anti-β-actin, anti-γ-actin and anti-GAPDH (loading control) antibodies. **(C)** Histogram showing the relative protein expression γ-actin as determined by densitometry after normalization with GAPDH, following treatment with control (*white*) and γ-actin siRNA (*black*) for 72 h. *Columns*, means of at least four individual experiments; *bars*, SE. Statistics were calculated by comparing γ-actin expression level in control and γ-actin siRNA-treated cells; ***, p < 0.001.

Significant knockdown of β-actin expression could not be achieved in endothelial cells without major cytotoxicity (data not shown). We therefore focused our attention on the functional analysis of γ-actin using RNA interference. As shown in Figure 2A, when HMEC-1 cells were transfected with γ-actin siRNA for 24 h, a 71 ± 12% reduction in γ-actin gene expression was observed by quantitative RT-PCR (p < 0.01). Consistently, a 50-60% knockdown of γ-actin expression was observed at the protein level after 72 h transfection in both HMEC-1 and BMH29L cells (Figure 2B and C; p < 0.001). Western blot analysis also showed that this level of γ-actin knockdown could be achieved without any significant compensatory changes in β-actin expression.

**Figure 3:**
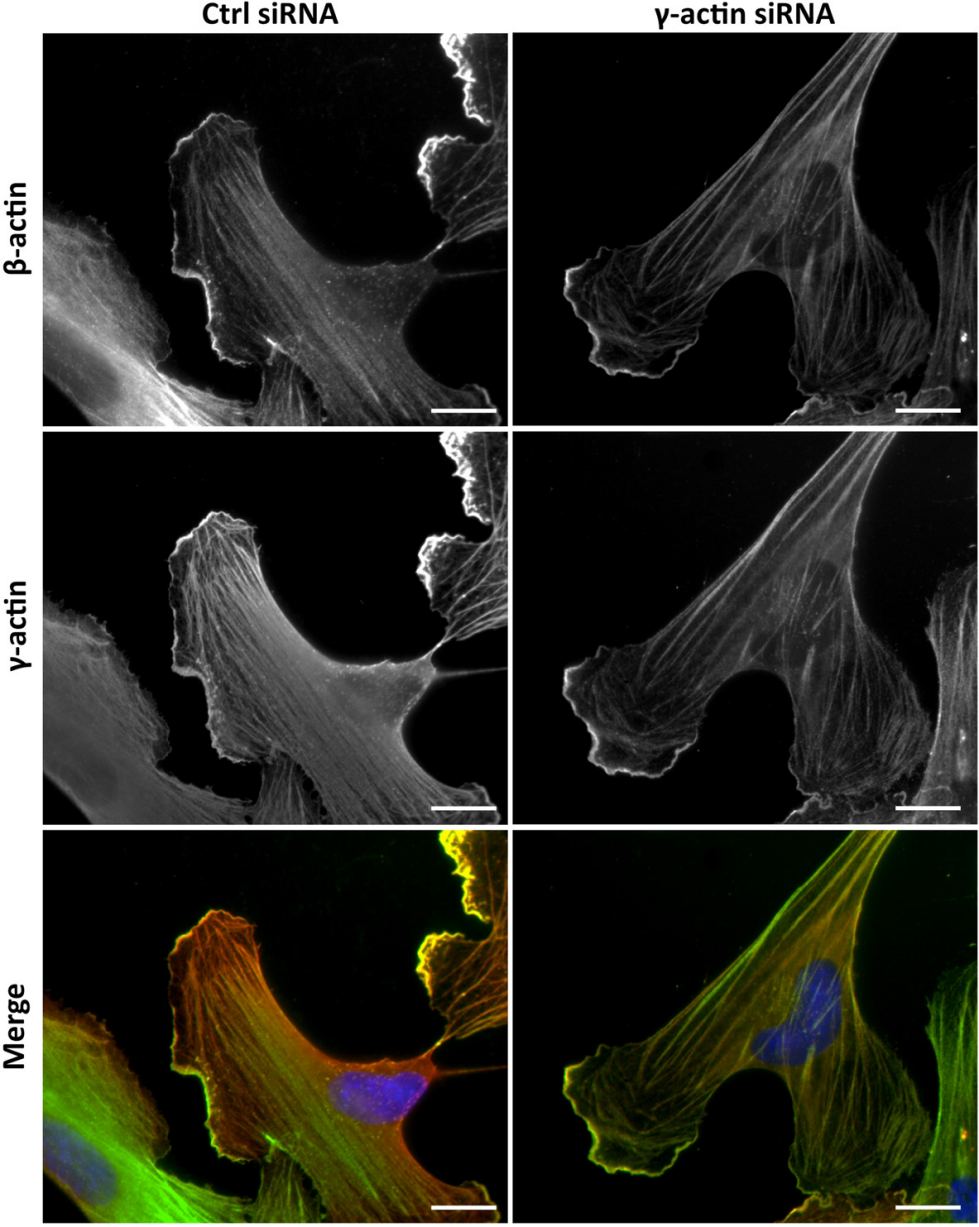
**Effect of** **γ-actin knockdown on the localization of** **β- and** **γ-actin.** Representative photographs of HMEC-1 endothelial cells treated for 72 h with control (*left*) or γ-actin siRNA (*right*) and stained with β-actin (*top*) and γ-actin (*middle*) antibodies. The merged photographs (*bottom*) show β-actin in green, γ-actin in red and DNA (DAPI) in blue. *Scale bar*, 20 μm.

Co-immunofluorescence staining was then used to analyse the effects of γ-actin knockdown on the localization of actin isoforms. In γ-actin siRNA-treated HMEC-1 cells, qualitative data revealed that the dense cortical actin meshwork was partially depleted in γ-actin and the remaining γ-actin was mostly found in stress fibres (Figure 3). In contrast, β-actin distribution was unaltered. Similar effects were observed in BMH29L cells (Additional file 2: Figure S2). Interestingly, quantitative analysis showed that γ-actin knockdown resulted in a slight increase in correlation of the β- and γ-actin signals, which was mostly due to decreased variation in stoichiometry (Additional file 1: Figure S1; Pearson coefficient of 0.75 ± 0.02 and 0.86 ± 0.01 in control and γ-actin siRNA-treated cells, respectively; p < 0.001).

### Cytoplasmic γ-actin expression is essential for the morphological differentiation of endothelial cells into vascular networks

**Figure 4:**
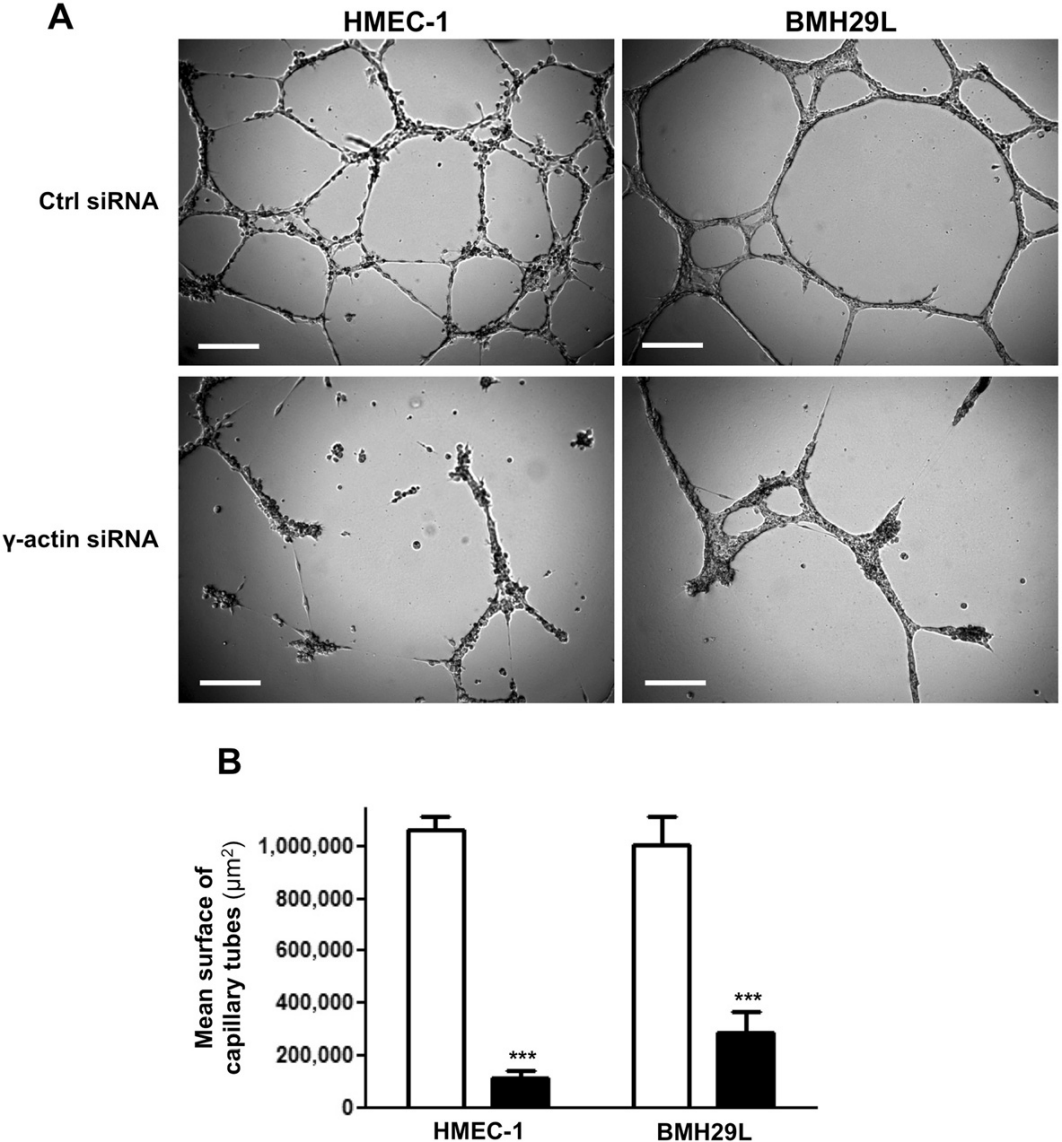
**Effect of** **γ-actin knockdown on the formation vascular networks** ***in vitro.*** **(A)** Representative photographs of HMEC-1 (*left*) and BMH29L cells (*right*) incubated for 8 h on Matrigel™. Cells were treated either with control (*top*) or γ-actin siRNA (*bottom*) for 72 h. *Scale bar*, 250 μm. **(B)** Histogram showing the surface occupied by vascular networks following treatment with control (*white*) and γ-actin siRNA (*black*) for 72 h. *Columns*, means of at least four individual experiments; *bars*, SE. Statistics were calculated by comparing the mean surface occupied by vascular networks per view field (at least 10 view fields per condition) for control siRNA- and γ-actin siRNA-treated cells. ***, p < 0.001.

Matrigel™ assay was used to investigate the role of γ-actin in angiogenesis *in vitro*. Photographs taken after 8 hour incubation on Matrigel™ revealed that knocking down γ-actin expression almost completely suppressed the formation of capillary-like structures (Figure 4A). Quantitative analysis showed that γ-actin knockdown inhibited the morphological differentiation of HMEC-1 and BMH29L cells by 89.5 ± 2.7% and 72.0 ± 6.7%, respectively (Figure 4B; p < 0.001). Similar results were obtained when HMEC-1 cells were transfected for 72 h with a different γ-actin siRNA sequence (Additional file 3: Figure S3). Time-lapse videomicroscopy experiments revealed that γ-actin siRNA-treated cells initiated the formation of capillary-like tubes on Matrigel™ but could not maintain these vascular networks, which rapidly regressed (Additional file 4: supplementary videos 1–2). This demonstrates that γ-actin is dispensable in the early steps of angiogenesis but required for neovessel maintenance.

### Vascular regression induced by γ-actin knockdown is associated with impaired VE-cadherin up-regulation

**Figure 5:**
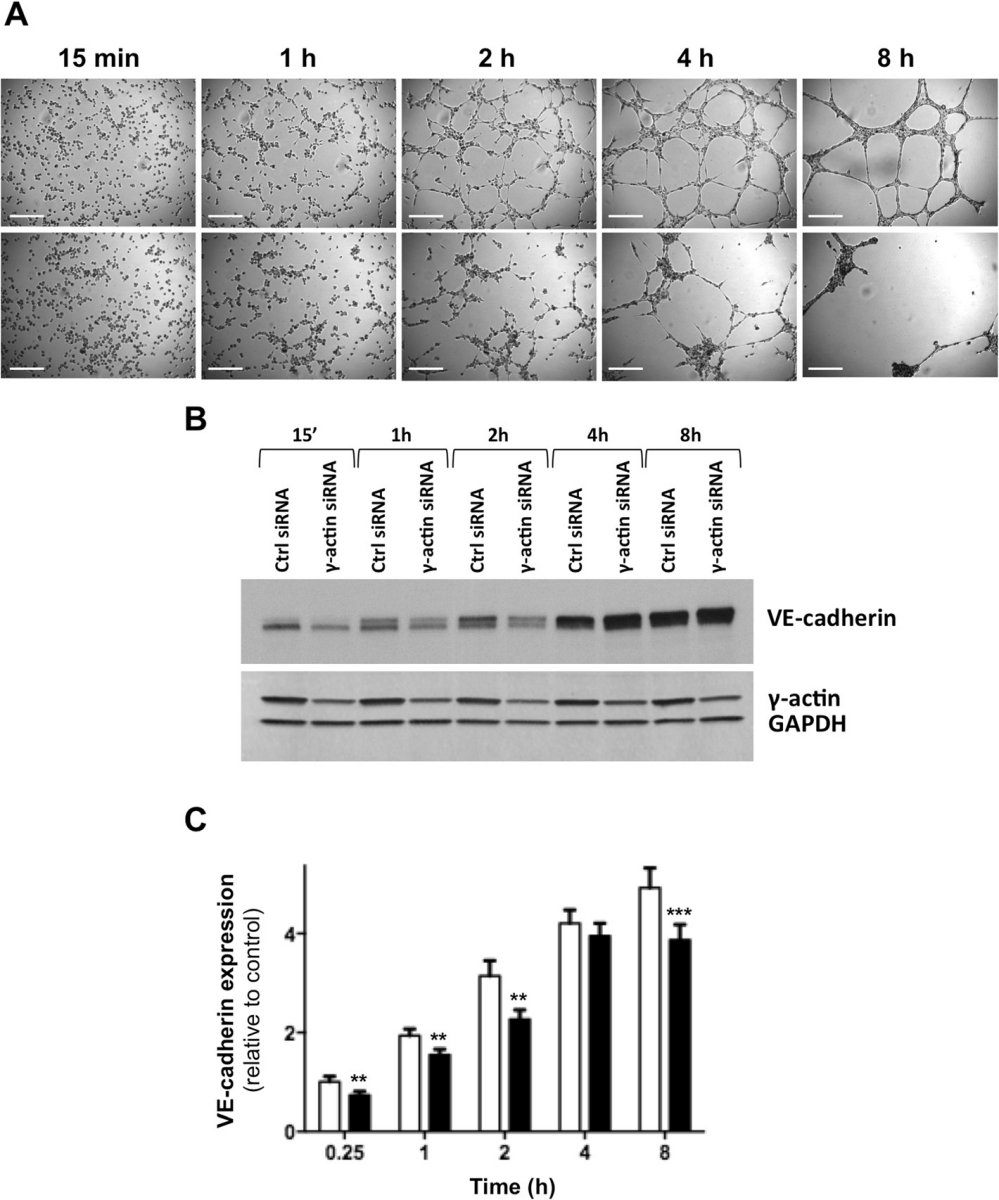
**Effect of** **γ-actin knockdown on VE-cadherin expression during morphological differentiation of endothelial cells into vascular networks. (A)** Representative photographs of HMEC-1 cells at various time points of the morphological differentiation process on Matrigel™, following treatment with control (*top*) and γ-actin siRNA (*bottom*) for 72 h. *Scale bar*, 250 μm. **(B)** Representative immunoblots of HMEC-1 cell lysates obtained at different time points of the morphological differentiation process on Matrigel™, following treatment with control and γ-actin siRNA for 72 h. Membranes were probed with anti-VE-cadherin, anti-γ-actin and anti-GAPDH (loading control) antibodies. **(C)** Histogram showing the relative protein expression of VE-cadherin as determined by densitometry after normalization with GAPDH (loading control), following treatment with control (*white*) and γ-actin siRNA (*black*) for 72 h. *Columns*, means of at least four individual experiments; *bars*, SE. Statistics were calculated by comparing VE-cadherin expression level in control and γ-actin siRNA-treated HMEC-1 cells; **, p < 0.01; ***, p < 0.001.

Endothelial cells were harvested non-enzymatically at different time points of the morphological differentiation process on Matrigel™ (Figure 5A) to investigate potential changes in expression of γ-actin, endothelial cell-to-cell contact protein VE-cadherin and major angiogenesis receptor VEGFR-2 by western blotting. While there was no significant change in γ-actin expression during the morphological differentiation of endothelial cells, VE-cadherin expression was gradually up-regulated (Figure 5B). Densitometry analysis revealed a 5-fold increase in VE-cadherin relative expression in control siRNA-treated cells between 15 min and 8 h incubation on Matrigel™ (Figure 5C; p < 0.001). Interestingly, γ-actin knockdown significantly impaired VE-cadherin up-regulation (Figure 5B and C). Although there was no significant difference in VE-cadherin expression between control siRNA- and γ-actin siRNA-treated cells at steady state when cells were grown on plastic (Additional file 5: Figure S4A), the levels of VE-cadherin expression were significantly lower in γ-actin siRNA-treated cells at all time points of the morphological differentiation process on Matrigel™ except at 4 h (Figure 5C). Additional western blot analysis further revealed that the expression of VEGFR-2 was also up-regulated during morphological differentiation, but this up-regulation was not affected by γ-actin knockdown (Additional file 5: Figure S4B). This suggests that the up-regulation of VE-cadherin expression, but not VEGFR-2, is dependent upon adequate γ-actin expression.

### Cytoplasmic γ-actin plays a key role in endothelial cell motility and chemotaxis

**Figure 6:**
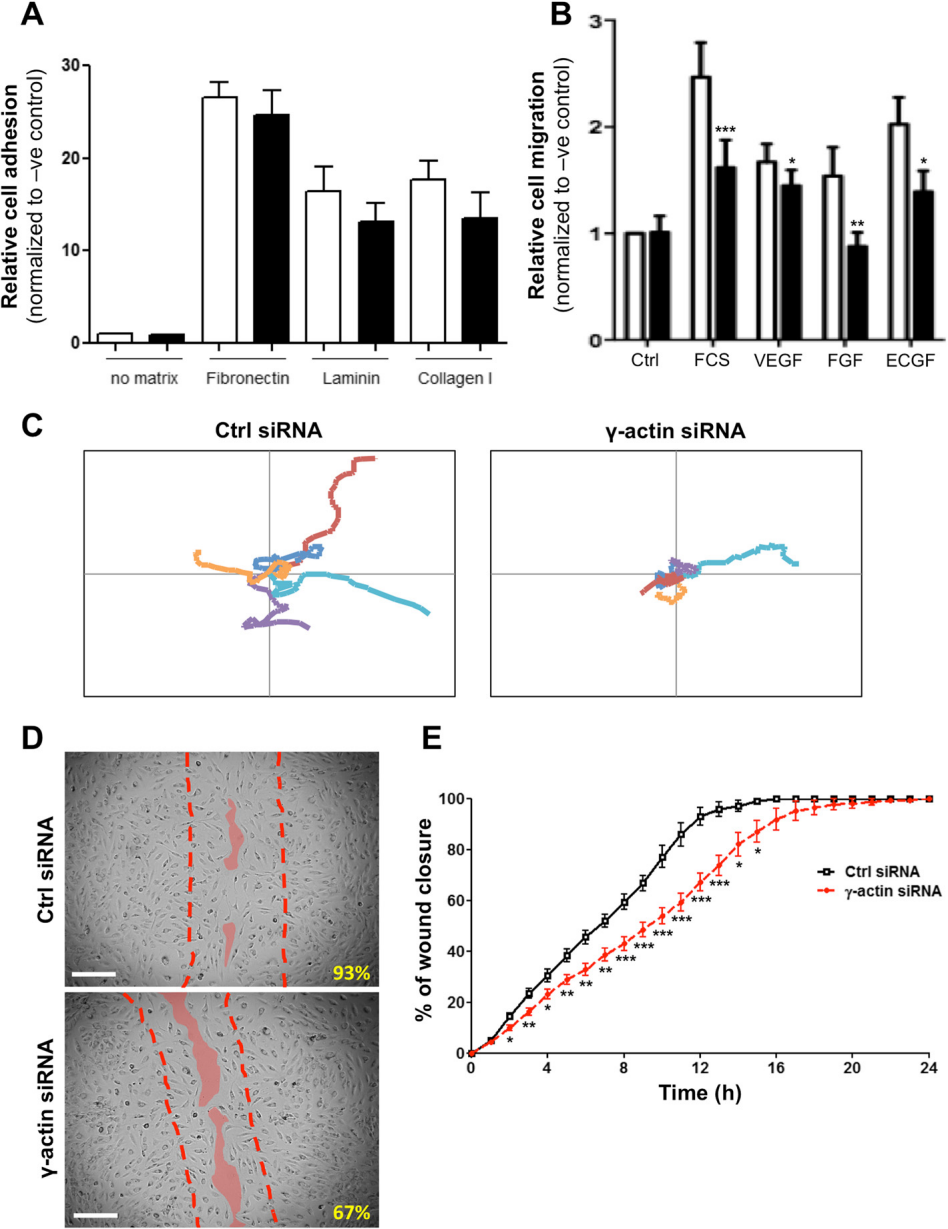
**Effect of** **γ-actin knockdown on cell adhesion, migration and motility. (A)** Histogram showing the relative adhesion of endothelial cells following treatment with control (*white*) and γ-actin siRNA (*black*) for 72 h. Fluorescently labeled HMEC-1 cells were allowed to adhere to various substrates for 1 h. *Columns*, means of at least three individual experiments; *bars*, SE. **(B)** Histogram showing the relative migration of endothelial cells towards FCS, VEGF, FGF_β_ and ECGF following treatment with control (*white*) and γ-actin siRNA (*black*) for 72 h. Fluorescently labeled HMEC-1 cells were allowed to migrate through 8 μm pore PET membrane and towards a chemo-attractant for 6 h. *Columns*, means of at least four individual experiments; *bars*, SE. Statistics were calculated by comparing the fluorescence measured at 492/517 (Abs/Em) with control and γ-actin siRNA-treated cells; *, p < 0.05; **, p < 0.001. **(C)** Representative trajectories of 5 individual HMEC-1 cells, recorded by time-lapse videomicroscopy over 6 h, following treatment with control (*left*) and γ-actin siRNA (*right*) for 72 h. *Scale*, −130 μm to +130 μm for both x and y axes. **(D)** Representative photographs of control (*top*) and γ-actin siRNA-treated (*bottom*) BMH29L cells in wound healing experiments, taken 12 h after start of experiment. Broken lines show the position of the initial cell-free gap (at time 0) and solid lines highlight the position of the migration edge after 12 h. *Inset*, % of wound closure. **(E)** Graph showing the percentage of wound recovery as a function of time for control (*black, solid line*) and γ-actin siRNA-treated (*red, broken line*) BMH29L cells. *Points*, means of at least four individual experiments; *bars*, SE. Statistics were calculated by comparing control and γ-actin siRNA-treated cells at specific time points; *, p < 0.05; **, p < 0.01; ***, p < 0.001.

To investigate the effects of γ-actin knockdown on the pro-angiogenic functions of endothelial cells, a panel of cell biology assays was used. First, we found that knocking down γ-actin expression did not significantly affect the adhesion of endothelial cells to various substrates, including major ECM proteins fibronectin, laminin and collagen I (Figure 6A). In contrast, knocking down γ-actin expression significantly impaired the chemotactic response of endothelial cells to various chemo-attractants. As shown in Figure 6B, addition of FCS, VEGF, FGF_β_ or ECGF in the bottom well of the Boyden chamber resulted in a significant increase in migration of control siRNA-treated HMEC-1 cells, as compared to the negative control (absence of FCS; p < 0.05). This increase in cell migration was significantly inhibited when HMEC-1 cells were transfected with γ-actin siRNA (p < 0.05). Interestingly, FGF_β_-induced migration was completely suppressed by γ-actin knockdown, while migration induced by other chemo-attractants was only partially reduced.

The effect of γ-actin knockdown on the random motility of endothelial cells was then assessed by time-lapse videomicroscopy experiments. Figure 6C shows the representative trajectories of 5 cells transfected with either control (*left panel*) or γ-actin siRNA (*right panel*). While control siRNA-treated cells randomly explored their micro-environment, γ-actin siRNA-treated cells appeared to stay focused around the same area. Analysis using the persistent random walk model [19] revealed that γ-actin knockdown decreased the average cell velocity and increased the time endothelial cells spent not moving as evidenced by an increase in persistence time (Table 1). As a result, γ-actin knockdown reduced the capacity of endothelial cells to explore their environment as shown by a decrease in random motility coefficient (i.e. the rate at which a cell will migrate into a new area and colonize it).

**Table 1:** **Effects of** γ**-actin knockdown on random motility parameters**

**Parameters**	**Control siRNA**	**γ-actin siRNA**	**% change**
Average cell velocity (μm/min)	0.53 ± 0.03	0.37 ± 0.03	−30 ± 5 %**
Persistence time (min)	6.3 ± 0.3	8.6 ± 0.6	+36 ± 7 %*
Random motility coefficient (μm^2^/min)	0.90 ± 0.05	0.55 ± 0.07	−38 ± 8 %**

At least 25 individual cells were analysed per view field using the persistence random-walk model [19]. Values are average of four individual experiments ± SEM. Statistics were calculated by comparing control and γ-actin siRNA-treated cells: *, p < 0.05; **, p < 0.01.

In order to further investigate the effects of γ-actin knockdown on endothelial cell migration, wound healing experiments were performed using time-lapse videomicroscopy. Figure 6D shows representative photographs taken from control (*top panel*) and γ-actin siRNA-treated cells (*bottom panel*) after 12 h incubation. At this time point, recovery from the wound was 93.0 ± 3.4% and 67.2 ± 3.5% in control and γ-actin siRNA-treated cells, respectively (p < 0.001). As shown in Figure 6E, γ-actin knockdown resulted in a significant decrease in wound recovery at all time points until 15 h. Linear regression analysis showed that control siRNA-treated BMH29L cells reached 50% wound recovery after 6.7 ± 0.4 h, whereas it took 9.4 ± 0.6 h (p < 0.01) to γ-actin siRNA-treated BMH29L cells (data not shown). Similar results were obtained with HMEC-1 cells, which took 7.8 ± 0.5 h and 9.8 ± 0.5 h to reach 50% wound recovery (p < 0.01), when they were transfected with control and γ-actin siRNA, respectively (data not shown).

### Cytoplasmic γ-actin regulates endothelial cell motility through ROCK signalling pathway

**Figure 7:**
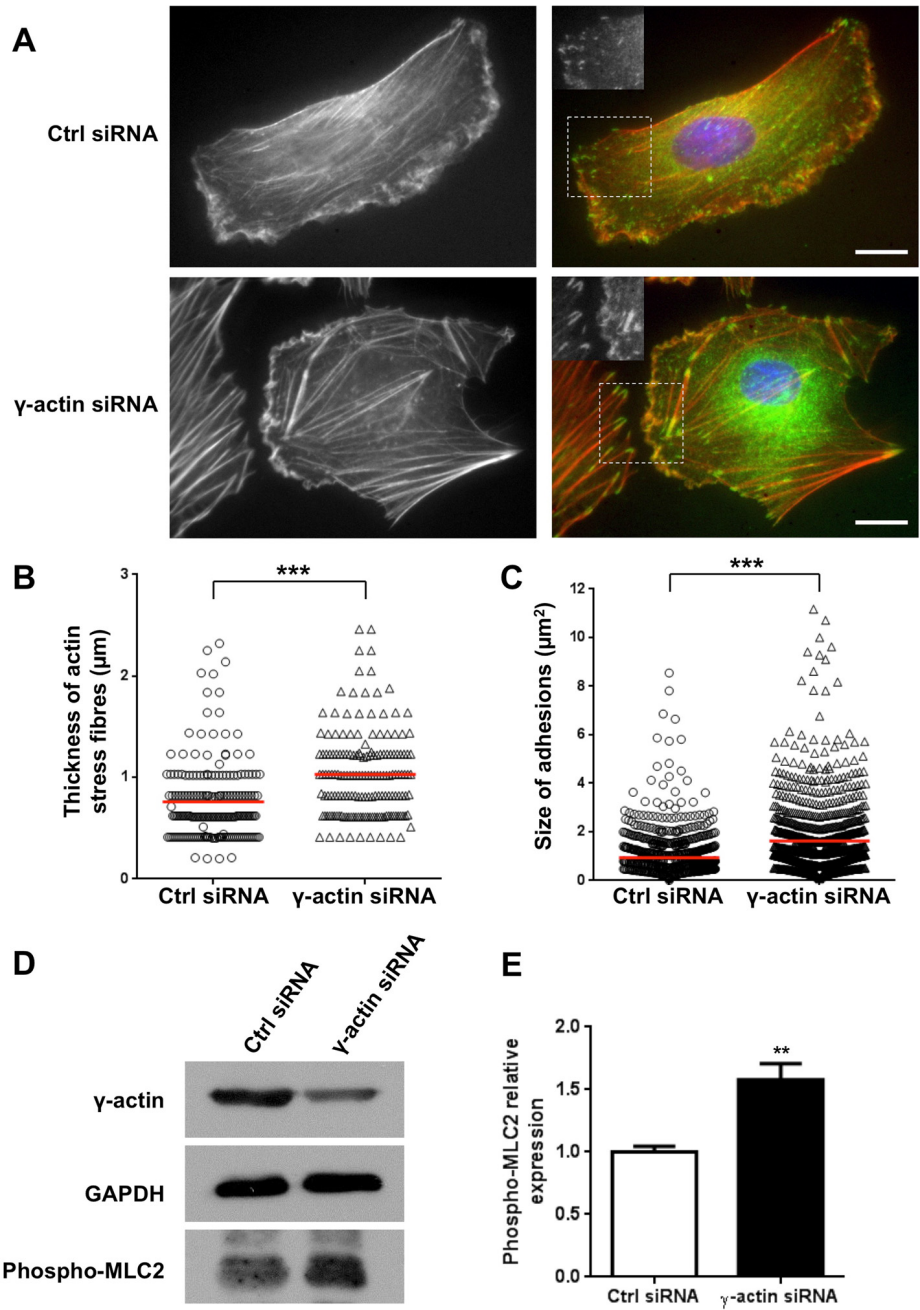
**Effect of** **γ-actin knockdown on ROCK signalling. (A)** Representative photographs of HMEC-1 endothelial cells treated for 72 h with control (*top*) or γ-actin siRNA (*bottom*) and co-stained with phalloidin (*left*) and anti-paxillin antibody. The merged photographs (*right*) show phalloidin in red, paxillin in green and DNA (DAPI) in blue. *Inset* shows a magnified view of paxillin staining in the lamellipodial region. *Scale bar*, 20 μm. **(B-C)** Scatter dot plots showing the thickness of actin stress fibres and the size of paxillin-containing adhesions in HMEC-1 endothelial cells treated for 72 h with control (o) or γ-actin siRNA (∆). *Bars*, means of at least 15 individual cells. **(D)** Representative immunoblots of HMEC-1 cell lysates following treatment with control and γ-actin siRNA for 72 h. Membranes were probed with anti-γ-actin, anti-GAPDH (loading control) and anti-phospho-myosin light chain 2 antibodies. **(E)** Histogram showing the relative levels of phosphorylated myosin light chain 2 in HMEC-1 cells following treatment with control (*white*) and γ-actin siRNA (*black*) for 72 h. *Columns*, means of at least four individual experiments; *bars*, SE. Statistics were calculated by comparing control siRNA- versus γ-actin siRNA-transfected cells. **, p < 0.01; ***, p < 0.001.

In order to gain more insights into the role of γ-actin in endothelial cell motility, we investigated potential changes in the organisation of microtubules, actin stress fibres and focal adhesions as a result of γ-actin knockdown. Tubulin staining of endothelial cells showed no significant change in the general organisation of the microtubule network as a result of γ-actin knockdown (Additional file 6: Figure S5). In contrast, co-immunofluorescence experiments with phalloidin and anti-paxillin antibody demonstrated that transfection of endothelial cells with γ-actin siRNA led to an accumulation of thick actin stress fibres and large paxillin-containing focal adhesions (Figure 7A). Quantitative analysis revealed that γ-actin knockdown induced a 38% increase in the average thickness of actin stress fibres, which was 0.75 ± 0.15 μm and 1.04 ± 0.17 μm in control and γ-actin siRNA-treated cells, respectively (Figure 7B; p < 0.001). This demonstrates that γ-actin knockdown results in a change in filamentous actin (F-actin) structures as a result of changing the β- to γ-actin ratio. Control siRNA-treated cells displayed uniform paxillin staining with small focal adhesions located throughout the cytoplasm and in the lamellipodia. In sharp contrast, γ-actin siRNA-treated cells showed larger focal adhesions dispersed throughout the cell periphery. Quantitative analysis showed that γ-actin knockdown did not alter the number of paxillin-containing adhesions but significantly increased their average size by 76%, from 0.96 ± 0.08 μm^2^ to 1.67 ± 0.11 μm^2^ (Figure 7C; p < 0.001). This accumulation of thick actin stress fibres and large focal adhesions could result from sustained activation of ROCK signalling [8]. Western blot analysis revealed that γ-actin knockdown was associated with a 58% increase in phosphorylation of myosin light chain 2 (Figure 7D-E), thus confirming activation of the ROCK pathway.

### Cytoplasmic γ-actin regulates angiogenesis through ROCK-dependent and ROCK-independent mechanisms

**Figure 8:**
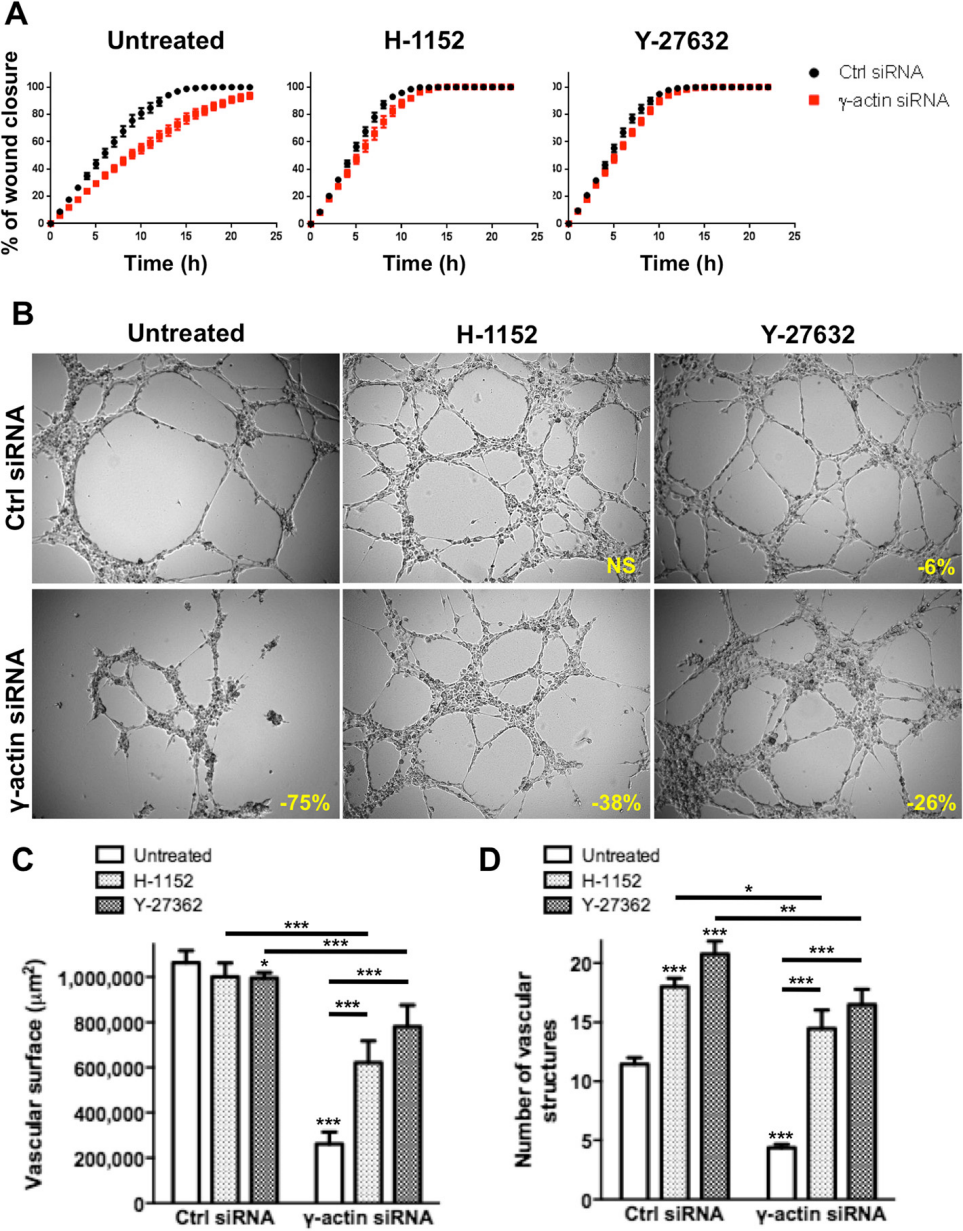
**Effect of ROCK signalling inhibition on** **γ-actin knockdown-induced vascular regression. (A)** Graph showing the percentage of wound recovery as a function of time for control (*black*) and γ-actin knockdown (*red*) HMEC-1 cells, either untreated (*left*) or treated with ROCK inhibitors, H-1152 (*middle*) or Y-27632 (*right*) at 1 and 10 μM, respectively. *Points*, means of at least four individual experiments; *bars*, SE. **(B)** Representative photographs of HMEC-1 cells incubated for 8 h on Matrigel^TM^. Cells were transfected with either control (*top*) or γ-actin siRNA (*bottom*) for 72 h and either untreated (*left*) or treated with ROCK inhibitors, H-1152 (*middle*) or Y-27632 (*right*) at 1 and 10 μM, respectively. *Inset*, % of angiogenesis inhibition as compared to untreated control cells. *Scale bar*, 250 μm. **(C-D)** Histograms showing the surface occupied by and the number of vascular networks formed by control and γ-actin siRNA-transfected HMEC-1 cells following treatment with ROCK inhibitors. *Columns*, means of at least four individual experiments; *bars*, SE. Statistics were calculated by comparing the mean surface occupied by vascular networks and the mean number of vascular networks per view field (at least 10 view fields per condition) for control siRNA- versus γ-actin siRNA-transfected cells unless indicated otherwise. *, p < 0.05; **, p < 0.01; ***, p < 0.001.

Pharmacological inhibition of ROCK signalling showed that the impairment of motility induced by γ-actin knockdown was entirely dependent upon ROCK activation (Figure 8A). Indeed, incubation with H-1152 and Y-27632 inhibitors completely restored the motility of γ-actin siRNA-treated HMEC-1 cells. Similar results were obtained with BMH29L cells (Additional file 7: Figure S6). In contrast with the complete rescue of the γ-actin knockdown motility phenotype by ROCK inhibitors, the capacity of γ-actin siRNA-treated cells to form and maintain vascular networks on Matrigel™ was only partially restored by ROCK inhibition (Figure 8B-D). Incubation of control siRNA-treated HMEC-1 cells with H-1152 and Y-27362 increased vascular sprouting and therefore led to the formation of more but significantly smaller capillary-like tubes, resulting in a marginal decrease in total vascular surface (4-6% decrease as compared to control untreated cells). The angiogenic potential of γ-actin siRNA-treated cells was improved when ROCK signalling was inhibited but vascular regression was not entirely prevented (Figure 8B). In γ-actin knockdown HMEC-1 cells, angiogenesis inhibition was thus reduced from 75 ± 6% in untreated cells to 38 ± 8% and 26 ± 8% in cells incubated with H-1152 and Y-27632, respectively (Figure 8C; p < 0.001). Quantification of the number of vascular networks formed confirmed that incubation with H-1152 and Y-27362 increased vascular sprouting (Figure 8D; p < 0.001) but γ-actin knockdown still resulted in significantly less networks formed in all conditions. Partial rescue of the angiogenesis suppression induced by γ-actin knockdown was also observed in BMH29L cells as a result of ROCK inhibition (Additional file 8: Figure S7). Collectively, the results of our study show that γ-actin plays a key role in the morphological differentiation of endothelial cells into vascular networks, through both ROCK-dependent and ROCK-independent mechanisms.

## Discussion

Actin-binding and regulatory proteins have been proposed to represent attractive targets for the development of innovative anti-angiogenic therapies [22]. However, the role of the actin cytoskeleton in angiogenesis has not been completely elucidated. Basic cell biology studies are crucially needed to map the specific role played by each component of the actin cytoskeleton in order to find new ways to target angiogenesis in pathologies such as cancer and rheumatoid arthritis. Here, we investigated the cellular distribution and localization of the two cytoplasmic isoforms of actin, β and γ, in vascular endothelial cells and performed the first functional analysis of γ-actin in these cells, revealing a key role for this protein in angiogenesis.

Co-immunofluorescence staining using newly developed antibodies directed against β- and γ-actin revealed a strong colocalization of the two cytoplasmic actin isoforms in vascular endothelial cells, albeit some level of spatial preference. Our findings are in accordance with the preferential localization of β-actin in radial stress fibres and membrane ruffling and γ-actin in the cortical microfilament meshwork previously reported in epithelial cells, fibroblasts [7] and more recently in neuroblastoma cells [8]. The subcellular localization of β- and γ-actin expression appears to be mediated by localization of their respective mRNA [5]. Furthermore, studies have shown that β- and γ-actin differentially interact with accessory proteins such as tropomyosins [23] and non-sarcomeric myosins [24]. These differential interactions may govern the specific functions of β- and γ-actin, and therefore contribute to the spatial and temporal regulation of cytoskeletal dynamics.

Significant knockdown of β-actin could not be achieved in vascular endothelial cells without inducing major cytotoxicity, suggesting that these cells are particularly sensitive to changes in β-actin expression. In contrast, γ-actin expression could be successfully knocked down by 50-60% in endothelial cells, indicating an isoform-specific function for actin in endothelial cells. This difference in response to β- and γ-actin knockdown is consistent with recent studies showing that β-actin knockout mice are not viable [9] unlike γ-actin knockout mice, which despite suffering increased mortality and showing progressive hearing loss during adulthood, are viable [10].

Interestingly, γ-actin knockdown almost completely suppressed the capacity of endothelial cells to maintain capillary-like structures on Matrigel™. Timelapse videomicroscopy analysis revealed that γ-actin was dispensable in the early steps of the morphological differentiation process (i.e. vascular sprouting) but required to sustain newly formed vascular networks. Previous studies have shown that the major endothelial cell adhesion molecule, VE-cadherin, plays a crucial role in angiogenesis – reviewed in [25-27]. The development of a VE-cadherin knockout (*Cdh5−/−*) mouse model thus revealed that this type II cadherin is dispensable in *de novo* blood vessel formation but essential to prevent the regression and ensure the maturation of nascent blood vessels [28,29]. We therefore hypothesized that VE-cadherin may be involved in the vascular regression induced by γ-actin knockdown. Our results showed that the expression of VE-cadherin is rapidly up-regulated during the morphological differentiation of endothelial cells, which is consistent with the study by Prandini *et al.* demonstrating that VE-cadherin promoter activity is enhanced during angiogenesis [30]. Furthermore, we found that the up-regulation of VE-cadherin was impaired in γ-actin siRNA-treated cells, which may contribute to vascular regression in these cells. This finding is in agreement with a recent study showing that VE-cadherin is involved in the cessation of endothelial cell sprouting [31]. Elsewhere, recent studies have shown that the major receptor involved in angiogenesis, VEGFR-2, may be in part regulated by the actin cytoskeleton. For instance, its localization to the plasma membrane has been shown to be mediated by myosin 1C, an unconventional motor protein trafficking along actin filaments [32]. In addition, inhibition of Rho GTPase Rac1 was found to down-regulate the expression VEGFR-2 [33]. However, we demonstrated herein that VEGFR-2 expression was up-regulated in endothelial cells during the angiogenic process independently of γ-actin expression.

To better understand how γ-actin knockdown impaired the angiogenic process, we investigated its impact on endothelial cell adhesion, chemotaxis and motility. While partially depleting γ-actin expression did not alter cell adhesion, it significantly reduced the capacity of endothelial cells to respond to chemotactic stimuli, randomly explore their microenvironment and colonize a new area. This result is in line with other studies demonstrating the crucial role played by the actin cytoskeleton in cell locomotion in non-endothelial cells – reviewed by Le Clainche and Carlier [34]. It is also consistent with the results recently reported for γ-actin depleted epithelial cells [7], arguing in favour of a crucial role of γ-actin in the control of directional motility in diverse cell types.

We previously reported that γ-actin regulates neuroblastoma cell migration by modulating ROCK signalling pathway [8]. Here, we extended this finding to vascular endothelial cells. Indeed, γ-actin knockdown was associated with accumulation of thicker actin stress fibres, larger focal adhesions and increased phosphorylation of myosin light chain 2 in endothelial cells, thus strongly suggesting activation of the ROCK signalling pathway. Furthermore, incubation with pharmacological inhibitors of ROCK signalling completely restored the motility of γ-actin siRNA-treated cells, demonstrating that the γ-actin knockdown motility phenotype is dependent upon ROCK activation. In contrast, ROCK inhibitors only partially rescued the angiogenic potential of γ-actin knockdown cells, suggesting that γ-actin regulates angiogenesis through both ROCK-dependent and ROCK-independent mechanisms.

Interestingly, VE-cadherin engagement has been shown to activate numerous biochemical pathways that can affect cell shape and adhesion, including the ROCK signalling pathway [35-37]. Furthermore, a direct link was recently reported between actin stress fibres and the protein complex forming adherens junctions, VE-cadherin/β-catenin/α-catenin [38,39]. Here we provide evidence of the reverse relationship since decreasing the expression of γ-actin partially blocked the required up-regulation of VE-cadherin expression during morphological differentiation of endothelial cells. Additional experiments are currently underway to fully decipher the mechanisms involved in the crosstalk between γ-actin, ROCK signalling and VE-cadherin, which appears to play a critical role in the maintenance of newly formed vascular networks.

## Conclusions

Collectively our results demonstrate for the first time that γ-actin plays a crucial role in angiogenesis through the regulation of endothelial cell motility and neovessel maintenance, *via* ROCK-dependent and -independent mechanisms. This finding opens potential therapeutic avenues for the treatment of angiogenesis-related disorders by targeting key factors involved in the γ-actin/VE-cadherin/ROCK signalling network.

## Additional files

Additional file 1: Figure S1. Quantification of β- and γ-actin colocalization. (A) Scatter dot plot showing the Pearson’s coefficient of individual HMEC-1 cells treated for 72 h with control (o) or γ-actin siRNA (∆). *Bars*, mean of three individual experiments with SE. Statistics were calculated by comparing control siRNA- versus γ-actin siRNA-transfected cells. ***, p < 0.001. (B) Representative 2-Dimensional histograms (fluorograms) showing a single linear relationship in high correlating samples (*left*) and multiple linear relationships in lower correlating cells (*right*). This shows that the lower correlation of β-actin and γ-actin signals in some cells was mostly due to variations in stoichiometry rather complete segregation of the two isoforms. Additional file 2: Figure S2. Effect of γ-actin knockdown on the actin cytoskeleton. Representative photographs of BMH29L endothelial cells treated for 72 h with control (*left*) or γ-actin siRNA (*right*) and stained with β-actin (*top*) and γ-actin (*middle*) antibodies. The merged photographs (*bottom*) show β-actin in green, γ-actin in red and DNA (DAPI) in blue. *Scale bar*, 20 μm. Additional file 3: Figure S3. Effect of γ-actin knockdown on the formation vascular networks on Matrigel™ using a different siRNA sequence. (A) Representative immunoblots of HMEC-1 cell lysates following treatment with control and γ-actin siRNA2 for 72 h. Membranes were probed with anti-β-actin, anti-γ-actin and anti-GAPDH (loading control) antibodies. (B) Histogram showing the relative protein expression of γ-actin as determined by densitometry after normalization with GAPDH (loading control), following treatment with control (*white*) and γ-actin siRNA2 (*black*) for 72 h. *Columns*, means of three individual experiments; *bars*, SE. Statistics were calculated by comparing γ-actin expression level in control and γ-actin siRNA2-treated HMEC-1 cells; ***, p < 0.001. (C) Representative photographs of HMEC-1 cells incubated for 8 h on Matrigel™ and following treatment with either control (*left*) or γ-actin siRNA (*bottom*) for 72 h. *Scale bar*, 250 μm. (D) Histogram showing the surface occupied by vascular networks following treatment with control (*white*) and γ-actin siRNA (*black*) for 72 h. *Columns*, means of three individual experiments; *bars*, SE. Statistics were calculated by comparing the mean surface occupied by vascular networks per view field (at least 10 view fields per condition) for control and γ-actin siRNA-treated HMEC-1 cells. ***, p < 0.001. Additional file 4:
**Supplementary videos 1–2.** Representative time-lapse videomicroscopy experiments of control siRNA- and γ-actin siRNA-treated endothelial cells on Matrigel™. BMH29L cells were seeded on Matrigel™ 72h after transfection with either control (Video 1) or γ-actin siRNA (Video 2). Photographs were taken every 5 min for 12 h, using the 5X objective of an Axiovert 200M fluorescent microscope coupled to an AxioCamMR3 camera driven by the AxioVision 4.7 software. Additional file 5: Figure S4. Effect of γ-actin knockdown on VE-cadherin and VEGFR-2 expression at steady state and during the angiogenic process. (A) Representative immunoblots of HMEC-1 (*left*) and BMH29L (*right*) cell lysates following treatment with control and γ-actin siRNA for 72 h. Membranes were probed with anti-VEGFR-2, anti-VE-cadherin, anti-γ-actin and anti-α-tubulin (loading control) antibodies. (B) Representative immunoblots of HMEC-1 cell lysates obtained at different time points of the morphological differentiation process on Matrigel™, following treatment with either control (*left*) or γ-actin siRNA (*right*) for 72 h. Membranes were probed with anti-VEGFR-2, anti-γ-actin and anti-GAPDH (loading control) antibodies. Additional file 6: Figure S5. Effect of γ-actin knockdown on the microtubule network. Representative photographs of HMEC-1 cells treated for 72 h with control (left) and γ-actin siRNA (right) and stained with anti-tubulin antibody. *Scale bar*, 20 μm. Additional file 7: Figure S6. Effect of ROCK signalling inhibition on γ-actin knockdown-induced motility inhibition. Representative photographs of wound healing experiments with control and γ-actin siRNA-transfected BMH29L cells. Endothelial cells were either untreated (*left*) or pre-treated with H-1152 (*middle*) or Y-27632 (*right*) at 10 μM for 24 hours and during the course of the experiment. Broken lines show the position of the initial cell-free gap (at time 0) and solid lines highlight the position of the migration edge after 8 h. *Inset*, % of wound closure. Additional file 8: Figure S7. Effect of ROCK signalling inhibition on γ-actin knockdown-induced vascular regression. (A) Representative photographs of BMH29L cells incubated for 8 h on Matrigel™. Cells were transfected with either control (*top*) or γ-actin siRNA (*bottom*) for 72 h and either untreated (*left*) or treated with 10 μM ROCK inhibitors, H-1152 (*middle*) or Y-27632 (*right*). *Inset*, % of angiogenesis inhibition as compared to untreated control cells. *Scale bar*, 250 μm. (B) Histogram showing the surface occupied by vascular networks following treatment with control (*white*) and γ-actin siRNA (*black*) for 72 h. *Columns*, means of at least four individual experiments; *bars*, SE. Statistics were calculated by comparing the mean surface occupied by vascular networks per view field (at least 10 view fields per condition) for control siRNA- vs γ-actin siRNA-transfected cells unless indicated otherwise. *, p<0.05; ***, p < 0.001.

## Abbreviations

ECM: Extra-cellular matrix
FCS: Fetal calf serum
FGF_β_: Fibroblast growth factor β
HMEC-1: Human microvascular endothelial cell line 1
MLC2: Myosin light chain 2
siRNA: Small interfering RNA
ROCK: Rho-associated protein kinase
VE-cadherin: Vascular endothelial cadherin
VEGFR-2: Vascular endothelial growth factor receptor 2
