# Supplementary figures and images for: γ-Actin plays a key role in endothelial cell motility and neovessel maintenance
Source: Vasc Cell. 2015 Feb 6;7:2. doi: 10.1186/s13221-014-0027-2 (PMC4335457; doi:10.1186/s13221-014-0027-2)

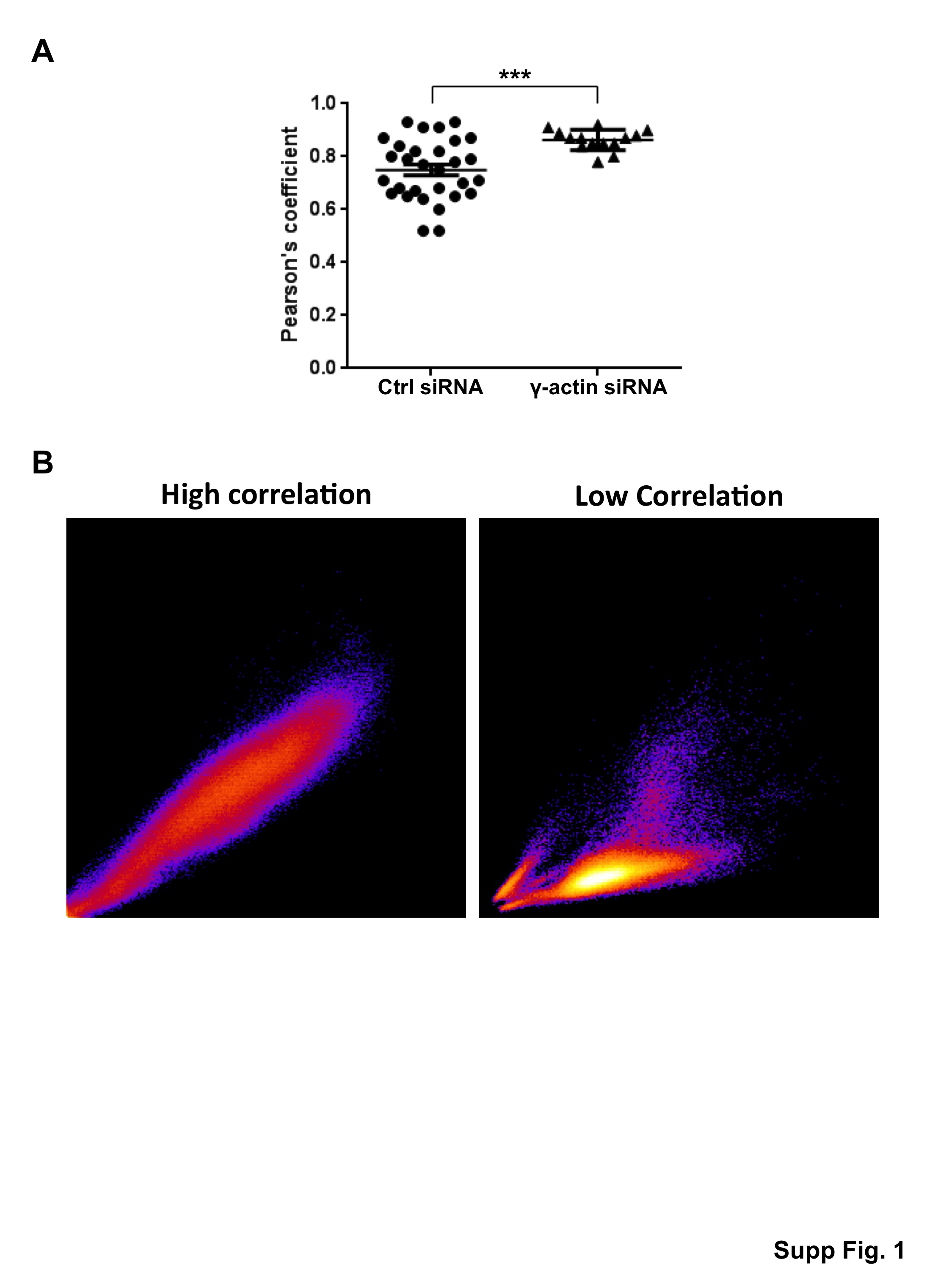

Supplement: Additional file 1: Figure S1. — Quantification of β- and γ-actin colocalization. (A) Scatter dot plot showing the Pearson’s coefficient of individual HMEC-1 cells treated for 72 h with control (o) or γ-actin siRNA (∆). Bars, mean of three individual experiments with SE. Statistics were calculated by comparing control siRNA- versus γ-actin siRNA-transfected cells. ***, p < 0.001. (B) Representative 2-Dimensional histograms (fluorograms) showing a single linear relationship in high correlating samples (left) and multiple linear relationships in lower correlating cells (right). This shows that the lower correlation of β-actin and γ-actin signals in some cells was mostly due to variations in stoichiometry rather complete segregation of the two isoforms. [file 13221_2014_27_MOESM1_ESM.tiff]

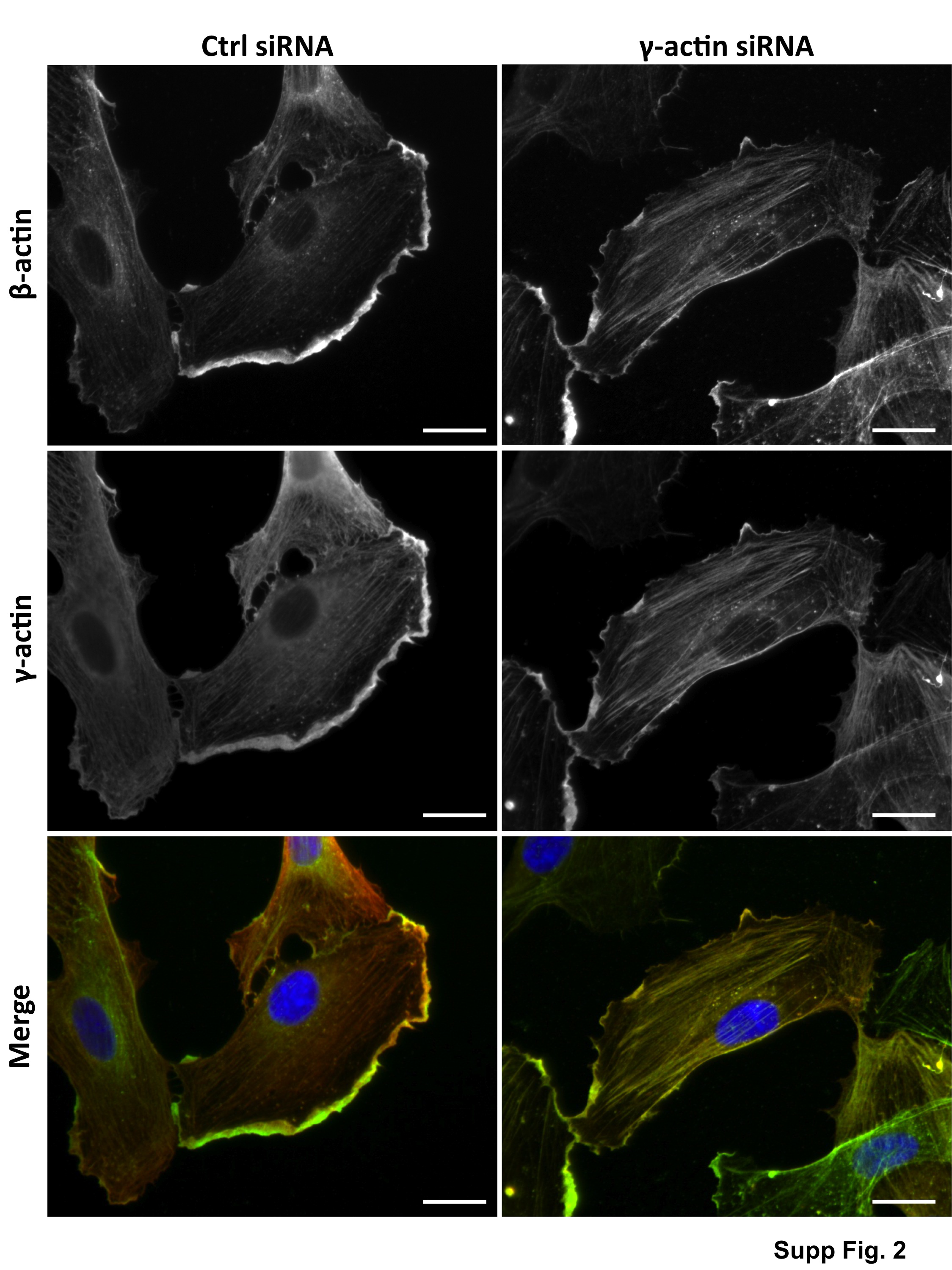

Supplement: Additional file 2: Figure S2. — Effect of γ-actin knockdown on the actin cytoskeleton. Representative photographs of BMH29L endothelial cells treated for 72 h with control (left) or γ-actin siRNA (right) and stained with β-actin (top) and γ-actin (middle) antibodies. The merged photographs (bottom) show β-actin in green, γ-actin in red and DNA (DAPI) in blue. Scale bar, 20 μm. [file 13221_2014_27_MOESM2_ESM.tiff]

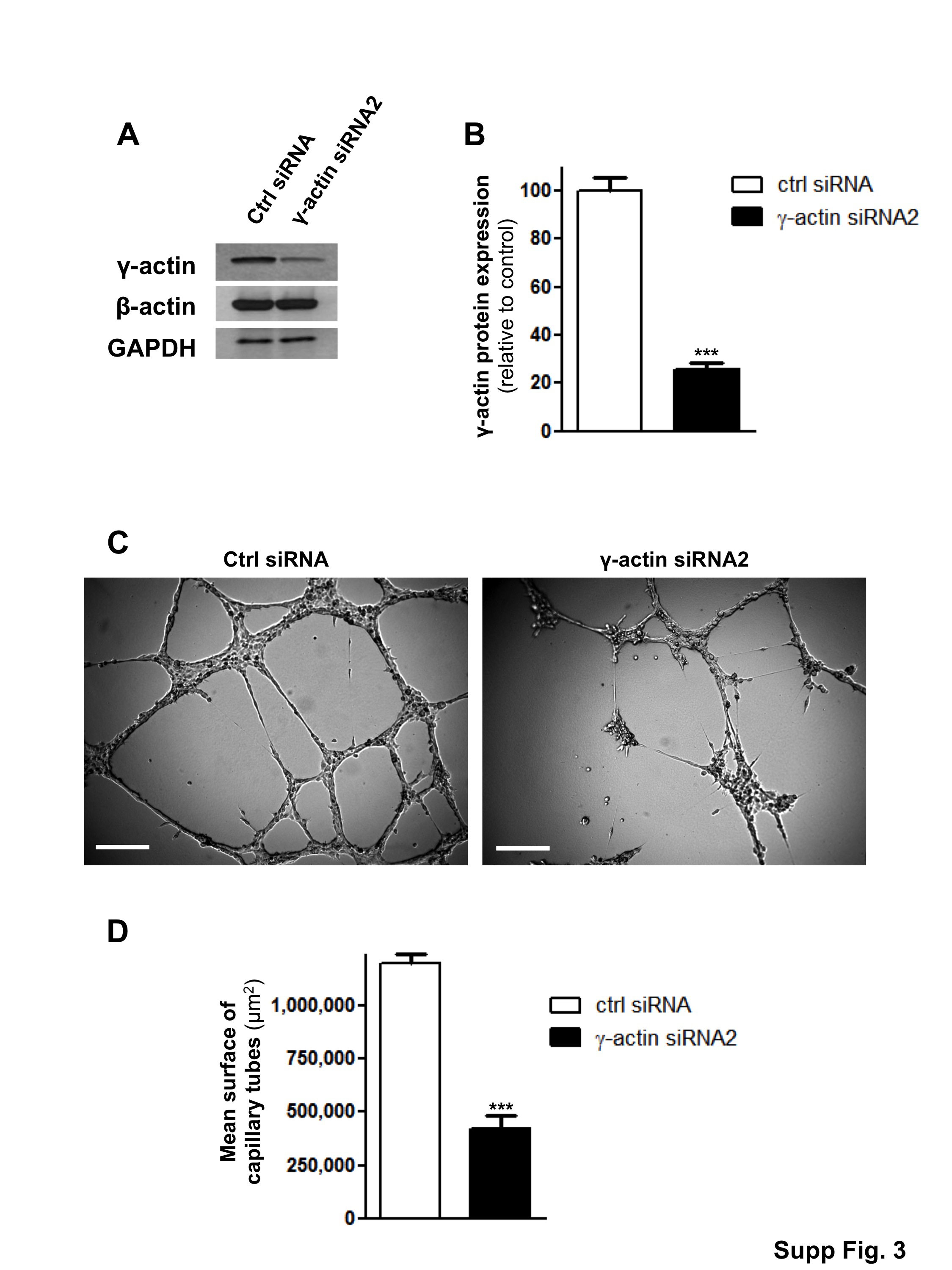

Supplement: Additional file 3: Figure S3. — Effect of γ-actin knockdown on the formation vascular networks on Matrigel™ using a different siRNA sequence. (A) Representative immunoblots of HMEC-1 cell lysates following treatment with control and γ-actin siRNA2 for 72 h. Membranes were probed with anti-β-actin, anti-γ-actin and anti-GAPDH (loading control) antibodies. (B) Histogram showing the relative protein expression of γ-actin as determined by densitometry after normalization with GAPDH (loading control), following treatment with control (white) and γ-actin siRNA2 (black) for 72 h. Columns, means of three individual experiments; bars, SE. Statistics were calculated by comparing γ-actin expression level in control and γ-actin siRNA2-treated HMEC-1 cells; ***, p < 0.001. (C) Representative photographs of HMEC-1 cells incubated for 8 h on Matrigel™ and following treatment with either control (left) or γ-actin siRNA (bottom) for 72 h. Scale bar, 250 μm. (D) Histogram showing the surface occupied by vascular networks following treatment with control (white) and γ-actin siRNA (black) for 72 h. Columns, means of three individual experiments; bars, SE. Statistics were calculated by comparing the mean surface occupied by vascular networks per view field (at least 10 view fields per condition) for control and γ-actin siRNA-treated HMEC-1 cells. ***, p < 0.001. [file 13221_2014_27_MOESM3_ESM.tiff]

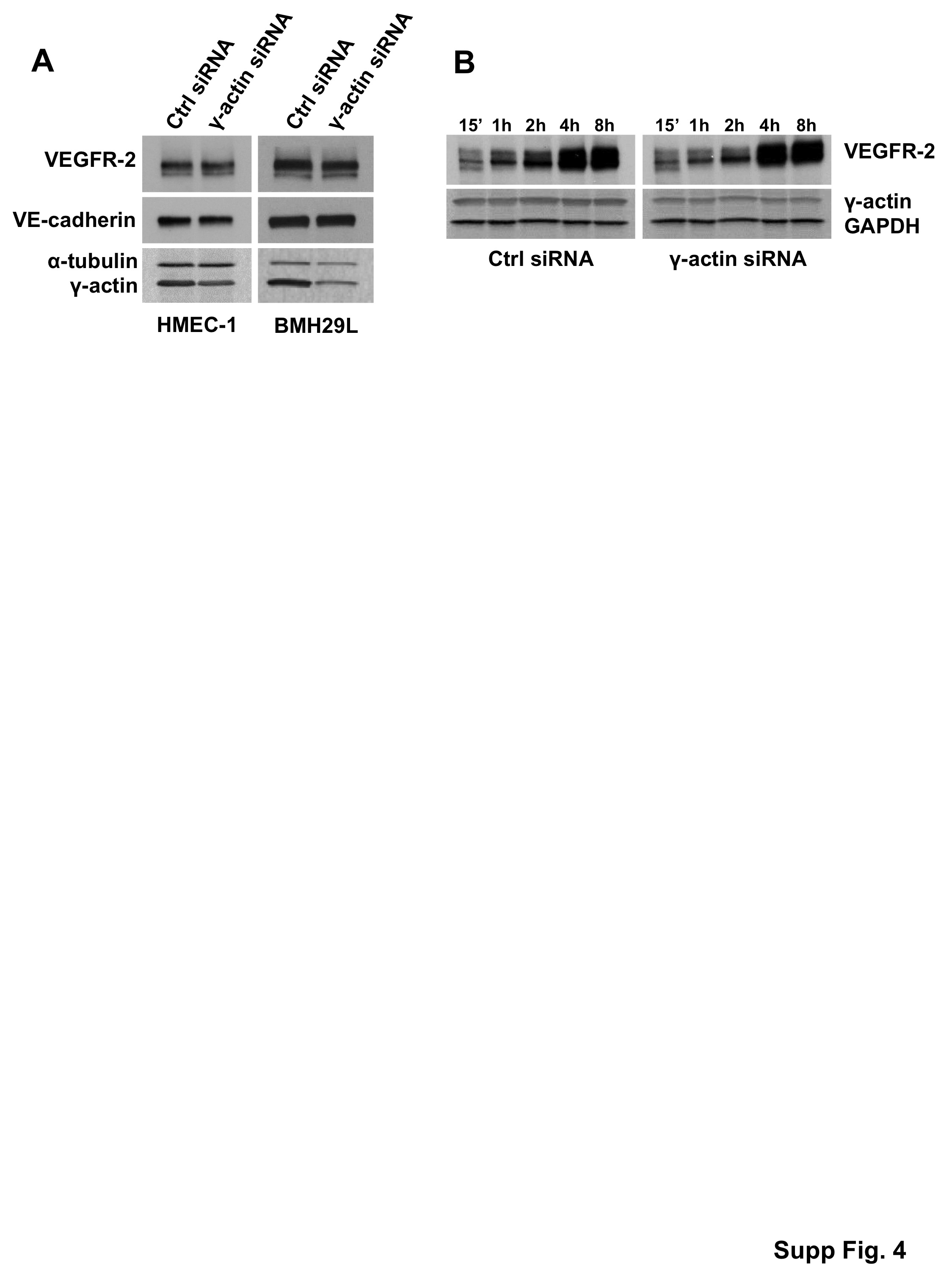

Supplement: Additional file 5: Figure S4. — Effect of γ-actin knockdown on VE-cadherin and VEGFR-2 expression at steady state and during the angiogenic process. (A) Representative immunoblots of HMEC-1 (left) and BMH29L (right) cell lysates following treatment with control and γ-actin siRNA for 72 h. Membranes were probed with anti-VEGFR-2, anti-VE-cadherin, anti-γ-actin and anti-α-tubulin (loading control) antibodies. (B) Representative immunoblots of HMEC-1 cell lysates obtained at different time points of the morphological differentiation process on Matrigel™, following treatment with either control (left) or γ-actin siRNA (right) for 72 h. Membranes were probed with anti-VEGFR-2, anti-γ-actin and anti-GAPDH (loading control) antibodies. [file 13221_2014_27_MOESM5_ESM.tiff]

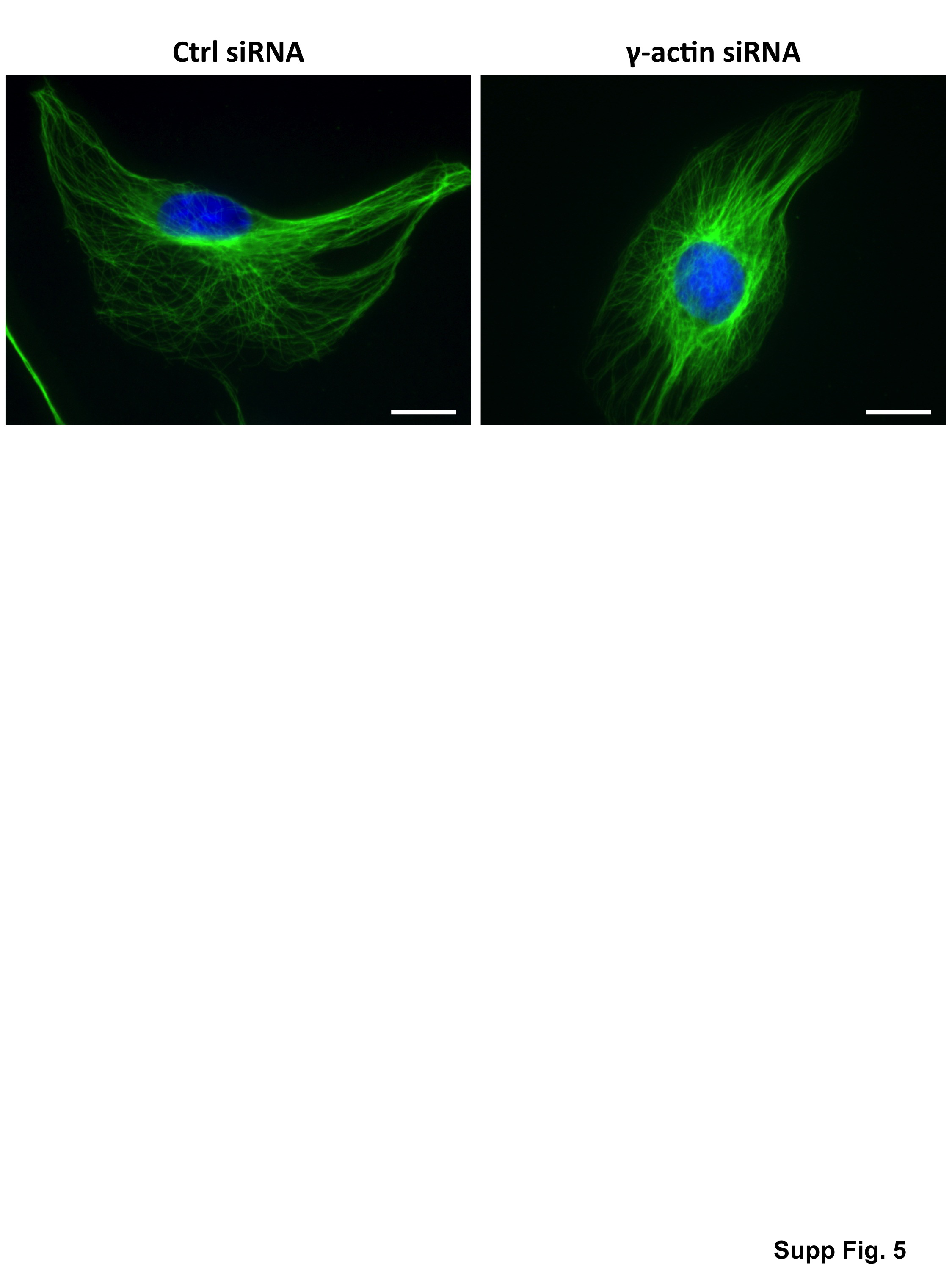

Supplement: Additional file 6: Figure S5. — Effect of γ-actin knockdown on the microtubule network. Representative photographs of HMEC-1 cells treated for 72 h with control (left) and γ-actin siRNA (right) and stained with anti-tubulin antibody. Scale bar, 20 μm. [file 13221_2014_27_MOESM6_ESM.tiff]

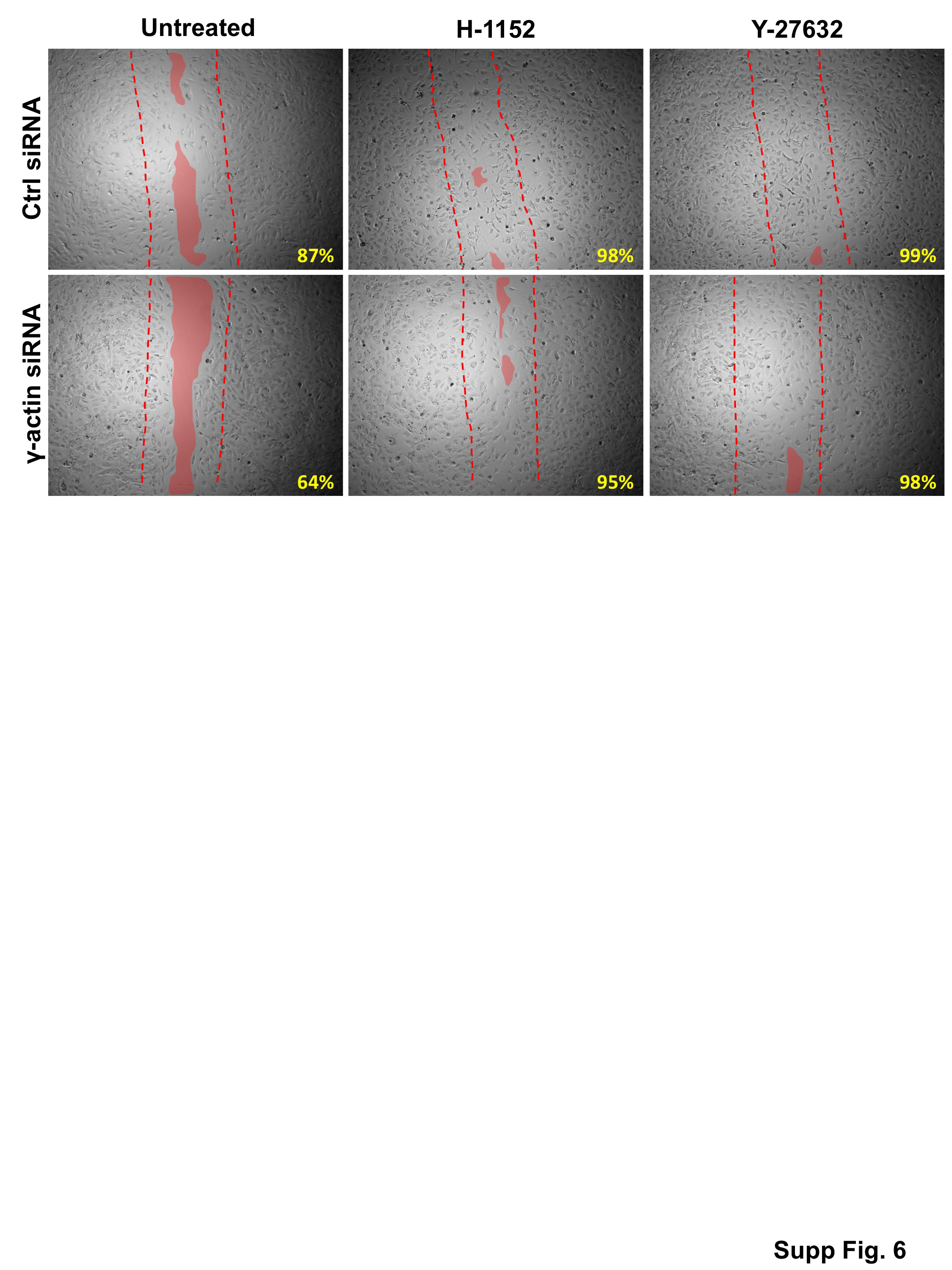

Supplement: Additional file 7: Figure S6. — Effect of ROCK signalling inhibition on γ-actin knockdown-induced motility inhibition. Representative photographs of wound healing experiments with control and γ-actin siRNA-transfected BMH29L cells. Endothelial cells were either untreated (left) or pre-treated with H-1152 (middle) or Y-27632 (right) at 10 μM for 24 hours and during the course of the experiment. Broken lines show the position of the initial cell-free gap (at time 0) and solid lines highlight the position of the migration edge after 8 h. Inset, % of wound closure. [file 13221_2014_27_MOESM7_ESM.tiff]

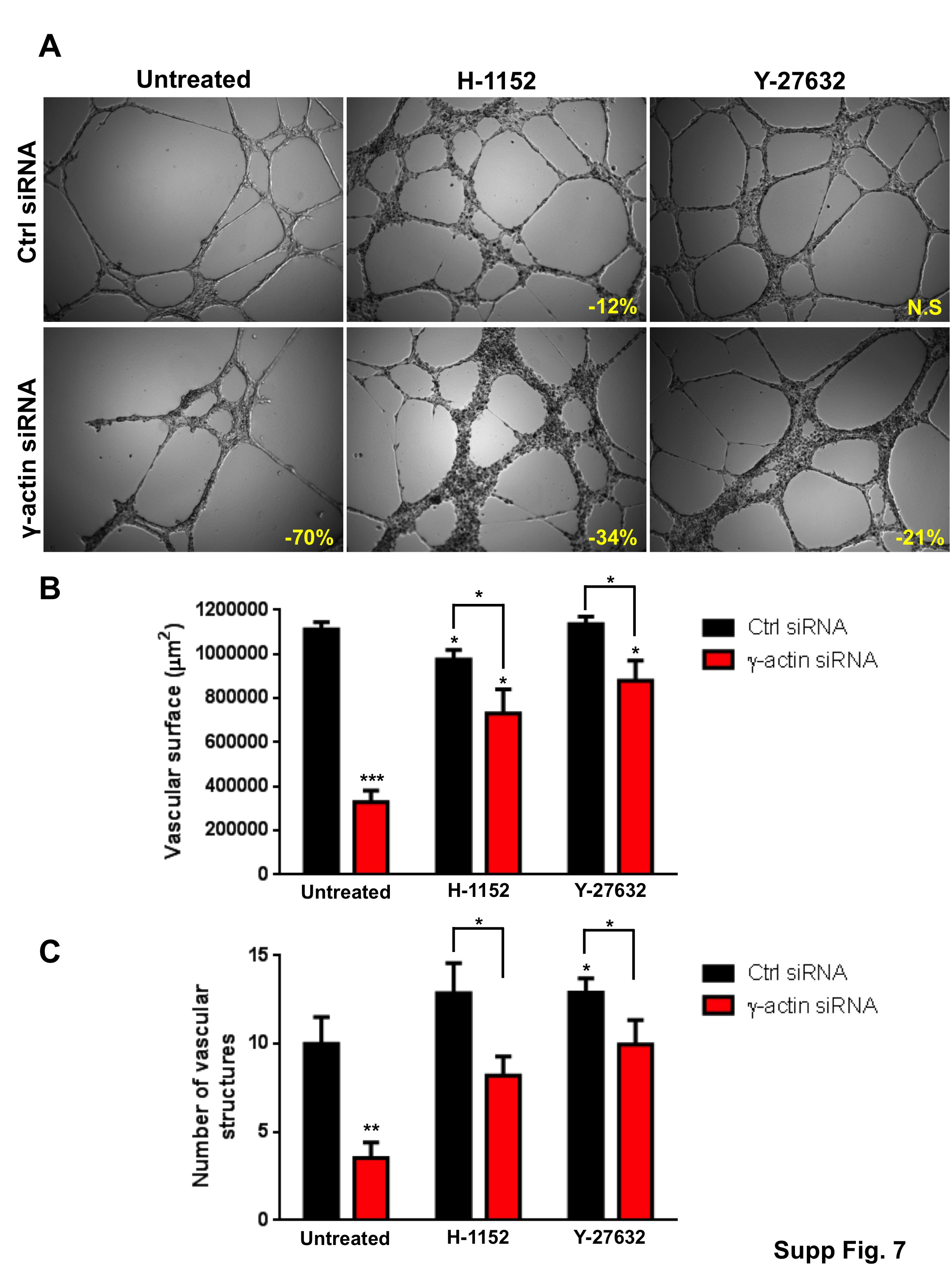

Supplement: Additional file 8: Figure S7. — Effect of ROCK signalling inhibition on γ-actin knockdown-induced vascular regression. (A) Representative photographs of BMH29L cells incubated for 8 h on Matrigel™. Cells were transfected with either control (top) or γ-actin siRNA (bottom) for 72 h and either untreated (left) or treated with 10 μM ROCK inhibitors, H-1152 (middle) or Y-27632 (right). Inset, % of angiogenesis inhibition as compared to untreated control cells. Scale bar, 250 μm. (B) Histogram showing the surface occupied by vascular networks following treatment with control (white) and γ-actin siRNA (black) for 72 h. Columns, means of at least four individual experiments; bars, SE. Statistics were calculated by comparing the mean surface occupied by vascular networks per view field (at least 10 view fields per condition) for control siRNA- vs γ-actin siRNA-transfected cells unless indicated otherwise. *, p<0.05; ***, p < 0.001. [file 13221_2014_27_MOESM8_ESM.tiff]
